# Overview of the Germline and Expressed Repertoires of the TRB Genes in *Sus scrofa*

**DOI:** 10.3389/fimmu.2018.02526

**Published:** 2018-11-05

**Authors:** Serafina Massari, Mariagrazia Bellini, Salvatrice Ciccarese, Rachele Antonacci

**Affiliations:** ^1^Department of Biological and Environmental Science and Technologies, University of Salento, Lecce, Italy; ^2^Department of Biology, University of Bari “Aldo Moro”, Bari, Italy

**Keywords:** T cell receptor, TRB locus, domestic pig, IMGT, TRB repertoire

## Abstract

The α/β T cell receptor (TR) is a complex heterodimer that recognizes antigenic peptides and binds to major histocompatibility complex (MH) molecules. Both α and β chains are encoded by different genes localized on two distinct chromosomal loci: TRA and TRB. The present study employed the recent release of the swine genome assembly to define the genomic organization of the TRB locus. According to the sequencing data, the pig TRB locus spans approximately 400 kb of genomic DNA and consists of 38 TRBV genes belonging to 24 subgroups located upstream of three in tandem TRBD-J-C clusters, which are followed by a TRBV gene in an inverted transcriptional orientation. Comparative analysis confirms that the general organization of the TRB locus is similar among mammalian species, but the number of germline TRBV genes varies greatly even between species belonging to the same order, determining the diversity and specificity of the immune response. However, sequence analysis of the TRB locus also suggests the presence of blocks of conserved homology in the genomic region across mammals. Furthermore, by analysing a public cDNA collection, we identified the usage pattern of the TRBV, TRBD, and TRBJ genes in the adult pig TRB repertoire, and we noted that the expressed TRBV repertoire seems to be broader and more diverse than the germline repertoire, in line with the presence of a high level of TRBV gene polymorphisms. Because the nucleotide differences seems to be principally concentrated in the CDR2 region, it is reasonable to presume that most T cell β-chain diversity can be related to polymorphisms in pig MH molecules. Domestic pigs represent a valuable animal model as they are even more anatomically, genetically and physiologically similar to humans than are mice. Therefore, present knowledge on the genomic organization of the pig TRB locus allows the collection of increased information on the basic aspects of the porcine immune system and contributes to filling the gaps left by rodent models.

## Introduction

In vertebrates, α/β T lymphocytes play central roles in the adaptive immune response. They recognize a large variety of foreign peptides bound to class I or class II major histocompatibility (MH) proteins. This recognition depends on the presence on the T cell membrane of a heterodimeric T cell receptor (TR) consisting of both an α and β chain. Each chain contains a variable and a constant domain. To generate a large repertoire of TR capable of recognizing diverse peptide–MH (pMH) complexes, during the development of the T lymphocytes in the thymus, the variable (V) and joining (J) genes of the TR α (TRA) locus and the V, diversity (D) and J genes of the TR β (TRB) locus undergo somatic rearrangements, with the resulting rearranged TRAV-TRAJ and TRBV-TRBD-TRBJ regions encoding the V-ALPHA and V-BETA variable domains of the α/β TR chains (1, 2). Each variable domain possesses three hypervariable regions termed complementarity determining regions (CDRs). CDR1 and CDR2 are encoded by the germline V gene and interact with MH molecules. A CDR3 loop is generated during the recombination process and is responsible for the interaction with antigen peptides. This implies that the number of the V, D and J genes in the germline DNA is an important element in generating the full extent of the TR repertoire. After transcription, the V-(D)-J sequence is spliced to the constant (C) gene.

The genomic organization of the TRB locus has been determined in representative species of several orders of mammals. A common feature of the genomic structure is a semi-cluster organization, with a pool of TRBV genes, numerically different from species to species, positioned upstream of in tandem aligned D-J-C clusters, each composed of a single TRBD gene, several TRBJ genes and one TRBC gene. A single inverted TRBV gene lies at the 3′ end of the last TRBC gene. In most mammals, i.e., human [ImMunoGeneTics (IMGT) Repertoire, http://www.imgt.org], dog (3) and rabbit (4), two TRBD-J-C clusters have been identified. In contrast, in the artiodactyl lineage (5–9), a duplication event within the 3′ end of the TRB locus has led to the generation of a third TRBD-J-C cluster, increasing the number of TRBD and TRBJ genes available for somatic recombination.

Partial information is available on the genomic organization of the pig TRB locus. As in the other artiodactyl species, the existence of three TRBD-J-C clusters in the pig genome was established by the analysis of two BAC clones representative of the 3′ portion of the TRB locus (6). Moreover, (10) reported a summary of porcine TRBV and TRBJ gene usage in thymocytes and peripheral T cells derived from a total of 329 PCR amplified clones. However, the lack of complete information on the overall structure of the porcine TRB locus limits the understanding of the results obtained by these efforts.

With the recent release of the swine genome assembly 11.1 (Sscrofa11.1) submitted by the Swine Genome Sequencing Consortium, we complete the genomic organization of the TRB locus and report an extensive analysis of the germline and expressed repertoires of the TRB genes in this species.

Domestic pigs are considered a valuable animal model because they are more closely related to humans than are mice in regard to their anatomy, genetics and physiology (11). The demand for pigs in immunological research has considerably increased over the last few years particularly for their potential use as organ donors in xenotransplantation but also for studies on various zoonotic infections that affect both pigs and humans (12, 13). Such work also requires a thorough understanding of the porcine immune system that has been shown to be very similar to its human counterpart in terms of anatomy, organization, and response (14, 15).

## Materials and methods

### Genome analyses

To determine the TRB locus location, the pig Sscrofa11.1 whole genome sequence, obtained by PacBio sequencing technology, was searched using the BLAST algorithm. A sequence of 402,496 bp was retrieved directly from the reference sequence NC_010460 (*Sus scrofa* chromosome 18 genomic scaffold) available at NCBI from 7348703 to 7741199 positions. In particular, the analyzed region comprises the MOXD2 and EPHB6 genes, already annotated within the scaffold and flanking respectively, the 5′ and 3′ ends of the TRB locus.

The entire human TRB genomic sequence and the available pig genomic sequence [(6), http://www.imgt.org, (16)] were used against the *Sus scrofa* genome sequence to identify, based on homology by the BLAST program, the corresponding genomic TRBV, TRBD, TRBJ and TRBC genes. Moreover, the homology-based method was used, aligning the pig retrieved sequence against itself with the PipMaker program (17) (Supplementary Figure S1). The beginning and end of each coding exon were identified with accuracy by the presence of splice sites or the flanking recombination signal sequences (RSs) of the TRBV, TRBD and TRBJ genes. The locations of the TRB genes are provided in Supplementary Table S1. The sequence comparison has also allowed for the identification and characterization of the pig protease genes (TRY). The locations of the TRY genes are provided in Supplementary Table S2.

The PipMaker program was also used for the genomic comparative analyses with the dog TRB genomic sequence previously described (3). Moreover, the computational analysis of the pig TRB locus was conducted using the RepeatMasker for the identification of genome-wide repeats and low complexity regions (Smit, A.F.A. Hubley, R. Green, P. RepeatMasker open-4.0. at http://www.repeatmasker.org).

### Classification of the Pig TRB genes

Considering the percentage of nucleotide identity of the genes with respect to human and the other mammalian species and based on the genomic position within the locus, each TRB gene was classified, and the nomenclature was established according to IMGT at http://www.imgt.org/IMGTScientificChart/SequenceDescription/IMGTfunctionality.html (18) (see Supplementary Table S1). The functionality of the V, D, J and C genes was predicted through the manual alignment of sequences adopting the following parameters: (a) identification of the leader sequence at the 5′ of the TRBV genes; (b) determination of proper RSs located at 3′ of the TRBV (V-RS), 5′ and 3′ ends of the TRBD (5′D-RS and 3′D-RS) and 5′ of the TRBJ (J-RS), respectively; (c) determination of conserved acceptor and donor splicing sites; (d) estimation of the expected length of the coding regions; (e) absence of frameshifts and stop codons in the coding regions of the genes.

The TRBV genes were assigned to 24 different subgroups, based on the percentage of nucleotide identity by using Clustal Omega alignment tool, which is available at EMBL-EBI website (http://www.ebi.ac.uk/), adopting the criterion that sequences with a nucleotide identity of more than 75% in the V-region belong to the same subgroup.

The TRBD, TRBJ and TRBC genes were annotated, according to the similarity with the other artiodactyl species (5, 8, 9). Each TRBJ1, TRBJ2, and TRBJ3 gene was designed by a hyphen and a number corresponding to their position in the cluster. They were all predicted to be functional except for the TRBJ1-4 (Supplementary Table S1).

### Phylogenetic analyses

The TRBV genes used for the phylogenetic analysis were retrieved from the following sequences deposited in the GEDI (for GenBank/ENA/DDBJ/IMGT/LIGM-DB) databases: NG_001333 (human TRB locus contig), NW_003726086 [dog TRB locus contig as characterized by 3); NW_011591622, NW_011593440, NW_011591151, NW_011620189, NW_011616084, NW_011607149, NW_011601111, and LT837971 [dromedary TRB locus contig as characterized by (8, 9)]; NC_010460 (pig TRB locus contig, this work).

The phylogenetic analysis was performed to classify the pig TRBV germline genes. We combined the nucleotide sequences of the V-REGION of the pig TRBV genes with the corresponding gene sequences of humans, dogs and dromedary.

Multiple alignments of the gene sequences under analysis were carried out with the MUSCLE program (19). Evolutionary analyses were conducted in MEGA7 (20). We used the neighbor-joining (NJ) method to reconstruct the phylogenetic tree (21). The evolutionary distances were computed using the p-distance method (22) and are in the units of the number of base differences per site.

The same phylogenetic method was also used to assign the TRBV4, TRBV5, TRBV7, and TRBV20 cDNA sequence collection to the germline TRBV genes and/or to classify new possible genes.

### cDNA analysis of public collection

Porcine cDNAs were retrieved from a collection submitted to public database (10) and were aligned with the germline TRB sequences determined in this study. From a total of 329 clones we have selected those derived from thymus and peripheral blood and obtained from different adult animals. 197 sequences, 35 from thymus (AY690915-AY690948 plus AY691000) and 166 from PBL (AY691001-AY691167), were analyzed in detail. One clone from thymus (AY690946) and three from PBL (AY691047, AY691092, and AY691114) have been eliminated since redundant.

The CDR3 size within the cDNA clones was calculated by the number of amino acids between the amino acid after the conserved 2nd cysteine in the V gene (pos.104), and the amino acid before the phenylalanina of the FGXG motif in the J gene [http://www.imgt.org, (18)].

## Results

### Analysis of the pig TRB locus retrieved from the genome assembly: identification of related and unrelated TRB genes

We employed the latest version of the whole genome assembly (Sscrofa11.1) of the pig (*Sus scrofa*) submitted by The Swine Genome Sequencing Consortium (SGSC) to NCBI (BioProject ID: PRJNA28993) to identify the TRB locus in this species. We retrieved from the pig whole chromosome 18 (chr18: 7348703–7741199) a sequence approximately 402 kb in length, comprising the MOXD2 and the EPHB6 genes that flank the 5′ and 3′ ends, respectively, of all mammalian TRB loci studied to date. The recovered sequence does not contain gaps as expected by the third-generation sequencing (23) First, we identified and annotated all TRB genes while considering as a reference both the human sequence [http://www.imgt.org/, (16)] and a continuous genomic sequence of 212 kb carried by two BAC clones and corresponding to the last portion of the porcine TRB locus (6). We also utilized a homology-based method, comparing the retrieved sequence with other mammalian corresponding regions (see the Genome analyses section in the “Materials and Methods”).

**Figure 1:**
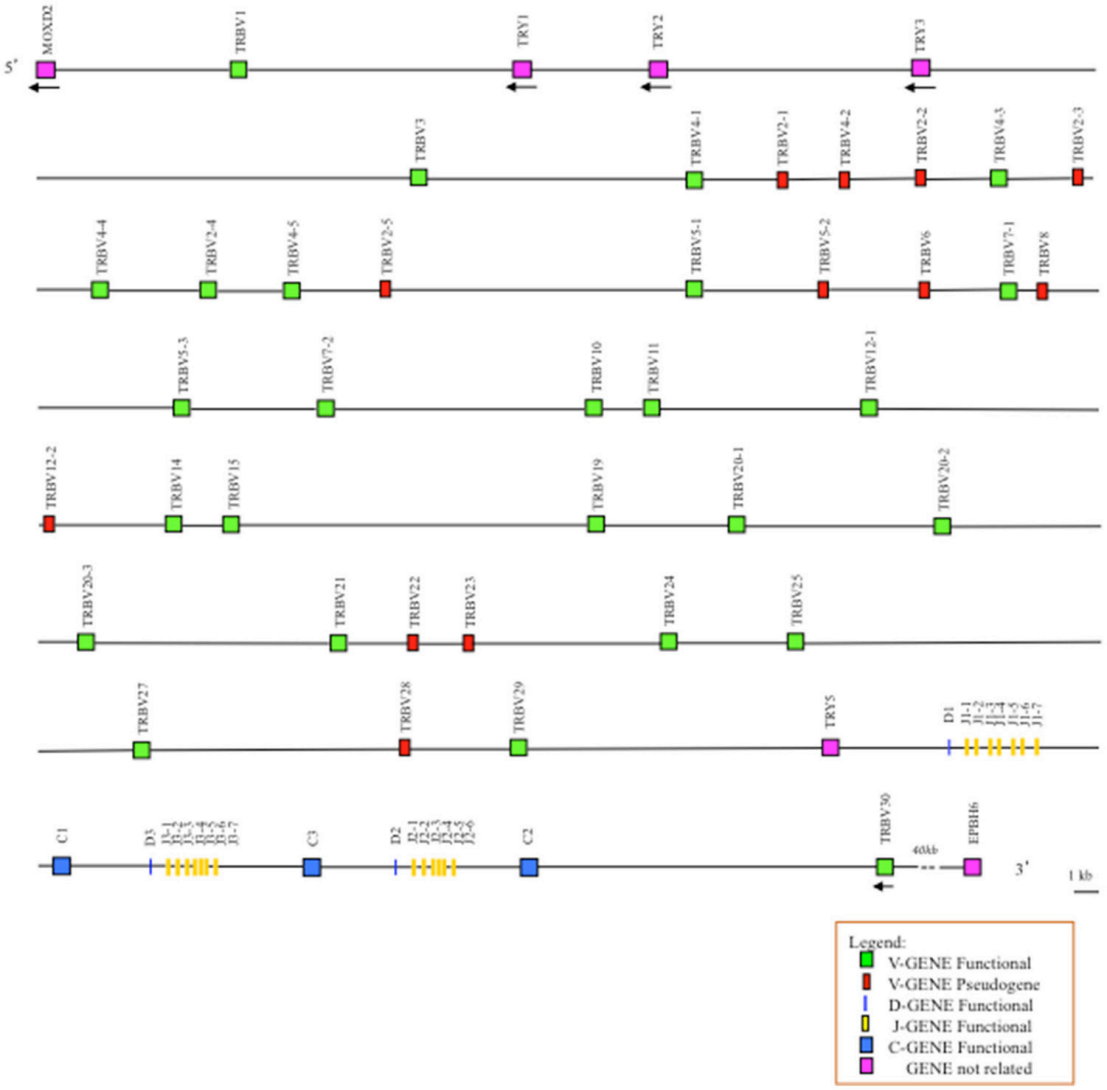
Schematic representation of the genomic organization of the pig TRB locus deduced from the genome assembly Sscrofa11.1. The diagram shows the position of all the related and unrelated TRB genes according to nomenclature. The boxes representing the genes are not to scale. The exons are not shown. The arrows indicate the transcriptional orientation of the genes.

An analysis of the genomic sequence revealed that the general structural organization of the pig TRB locus follows that of the other mammalian species, with a library of TRBV genes positioned at the 5′ end of D-J-C clusters, followed by a single TRBV gene located at the 3′ end in an inverted transcriptional orientation (Figure 1). Specifically, analysis of the assembly confirmed the presence of three D-J-C clusters similar to the clusters found in other species of artiodactyls, where D-J-C cluster 3 lies between D-J-C cluster 1 and D-J-C cluster 2 (5, 8, 9) The pig D-J-C region covers approximately 27 kb. D-J-C cluster 1 spans 7,659 bp and contains one TRBD, seven TRBJ and one TRBC genes. D-J-C cluster 3 is located at 3 kb both downstream of cluster 1 and upstream of cluster 2, with a total length of 7,296 bp, and includes one TRBD, seven TRBJ and one TRBC genes. Finally, D-J-C cluster 2 extends over 5,828 bp, with one TRBD, six TRBJ and one TRBC genes. Approximately 13 kb away from the TRBC2 gene lies the TRBV30 gene. The organization and nucleotide sequence identity of nine TRBV genes upstream of the D-J-C region as well as the D-J-C genes themselves in the assembly roughly match with that identified in the BAC clones (6). The classification, position and predicted functionality of all TRB genes are reported in Supplementary Table S1.

The comparison of the entire pig TRB sequence allowed us to identify and annotate four unrelated TRB genes consisting of a group of trypsin-like serine protease (TRY) genes that are typically interspersed among the mammalian TRB genes. They are named on the basis of the bovine corresponding gene positions (7). Three are located downstream of TRBV1, and one is located upstream of the D-J-C region (Figure 1). Their classification, position and predicted functionality are reported in Supplementary Table S2, together with those of the MOXD2 and EPHB6 genes, which delimit the TRB locus.

### Classification of the TRBV genes

We annotated 38 TRBV germline genes, which can be assigned to 24 distinct subgroups, adopting the criterion that sequences with a nucleotide identity of more than 75% in the V region belong to the same subgroup. Six subgroups are multimembers with prominent expansion of the TRBV2 and TRBV4 subgroups (five genes each). The TRBV5 and TRBV20 subgroups consist of three genes, while both the TRBV7 and TRBV12 subgroups contain two genes. TRBV2-2 and TRBV2- 3 display the same coding region, with a few nucleotide variations within their introns, while TRBV4-3 and TRBV4-4 exhibit identical sequences. Moreover, TRBV20-2 shows a high nucleotide identity (>97%) with the other two TRBV20 subgroup members (TRBV20-1 and TRBV20-3). Twenty-six out of 38 genes are predicted to be functional (approximately 68%) as defined by the IMGT rules (see the “Materials and Methods”), and 12 are pseudogenes (Supplementary Table S3).

**Figure 2:**
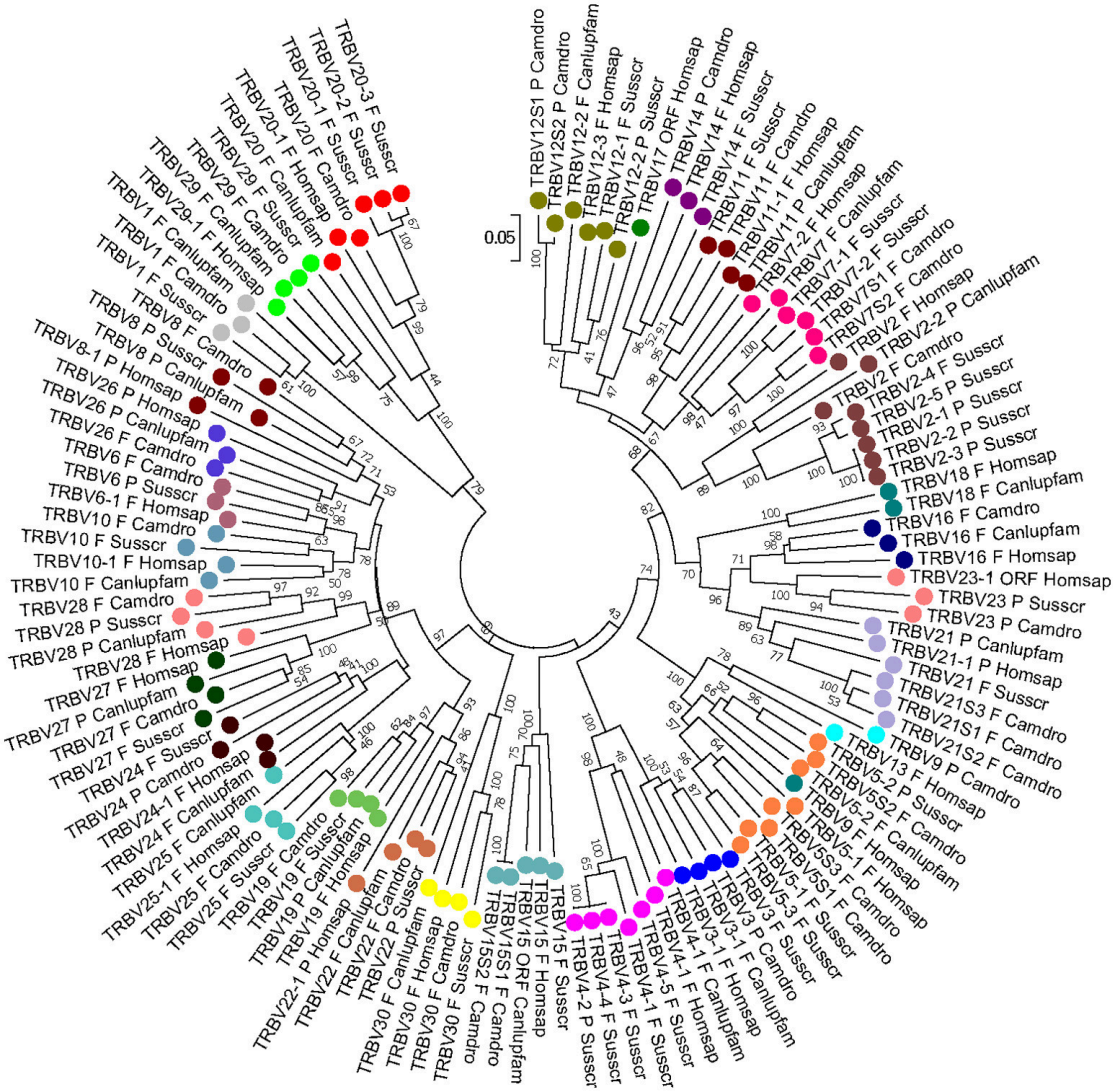
The NJ tree inferred from the pig, human, dog and dromedary TRBV gene sequences. The evolutionary analysis was conducted in MEGA7 (20). The optimal tree with the sum of branch length = 14.02591240 is shown. The percentage of replicate trees in which the associated taxa clustered together in the bootstrap test (1,000 replicates) is shown next to the branches (24). The tree is drawn to scale with branch lengths in the same units as those of the evolutionary distances used to infer phylogenetic trees. The evolutionary distances were computed using the p-distance method (22) and are in the units of the number of base differences per site. The analysis involved 124 nucleotide sequences. Codon positions included were 1st+2nd+3rd+Noncoding. All positions containing gaps and missing data were eliminated. There were a total of 163 positions in the final dataset. The pig TRBV subgroup classification is performed according to the clustering with the orthologous mammalian TRBV subgroups. The different colors highlight the distribution of the phylogenetic groups. The gene functionality according to IMGT rules (F: functional, ORF: open reading frame, P: pseudogene) is indicated. The IMGT 6-letter for species (Homsap, Camdro, Susscr) and 9-letter for subspecies (Canlupfam) standardized abbreviation for taxon is used.

To classify the pig TRBV gene subgroups, the evolutionary relationship of these genes was investigated by comparing all of the pig genes with available corresponding genes in human, dog and dromedary by adopting two selection criteria: (1) only potential functional genes and in-frame pseudogenes (except for human TRBV9 and TRBV1) were included; and (2) only one gene per each of the subgroups was selected for human and dog. Thus, the V-REGION nucleotide sequences of all selected TRBV genes were combined in the same alignment, and an unrooted phylogenetic tree was made using the NJ method (21) (Figure 2). The tree shows that each of the 24 pig subgroups form a monophyletic group, when present, with corresponding human, dog and dromedary genes, consistent with the occurrence of distinct subgroups prior to the divergence of the different mammalian species. Therefore, according to phylogenetic clustering, we classified each pig TRBV subgroup as orthologous to its corresponding mammalian subgroup.

Five human TRBV subgroups (TRBV13, TRBV16, TRBV17, TRBV18, and TRBV26) were not found in pig, with TRBV17 missing also in the dog and dromedary genomes; TRBV16 and TRBV18 were also lacking at the dromedary TRB locus, while TRBV13 is absent in the dog genome (3, 8, 9). Notably, human TRBV9 is grouped together with the orthologous TRBV5-2 genes of the other species, indicating that the classification of the human gene should be revised. Moreover, pig TRBV1 groups together with the corresponding dog and dromedary genes, while it is a pseudogene in the human genome (IMGT Repertoire, http://www.imgt.org).

**Figure 3:**
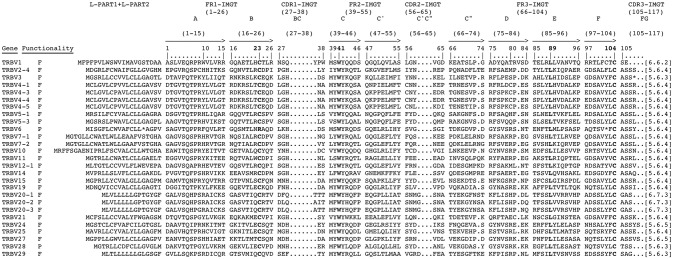
The IMGT Protein display of the pig TRBV genes. Only functional genes and in-frame pseudogenes are shown. The description of the strands and loops and of the FR-IMGT and CDR-IMGT is according to the IMGT unique numbering for V-REGION (25). The five conserved amino acids of the V-DOMAIN (1st-CYS 23, except for the TRBV6 pseudogene, CONSERVED-TRP 41, hydrophobic AA 89, 2nd-CYS 104 and J-PHE 118) are indicated in bold. The amino acid length of the CDR-IMGT AA is also indicated in square brackets.

The deduced amino acid sequences of the pig germline TRBV genes were manually aligned according to IMGT unique numbering for the V-REGION (25) to maximize the percentage of identity (Figure 3). Only potential functional genes and in-frame pseudogenes are shown. All sequences exhibit the typical framework regions (FR) and complementarity determining regions (CDR) as well as four amino acids: cysteine 23 (1st-CYS) in FR1-IMGT (except for the TRBV6 pseudogene), tryptophan 41 (CONSERVED-TRP) in FR2-IMGT, hydrophobic amino acid 89, and cysteine 104 (2nd-CYS) in FR3-IMGT (25). Conversely, CDR-IMGT varies in amino acid composition and length.

### Description and classification of the TRBD, TRBJ, and TRBC genes retrieved from the assembly

As in ruminant and dromedary species, (5, 7–9), the pig TRBD, TRBJ and TRBC genes were distributed within three in tandem D-J-C clusters positioned at the 3′ end of the TRB locus, with a number corresponding to their position from 5′ to 3′ (Figure 1). We have attributed the name D-J-C cluster 3 to the central cluster as in sheep and dromedary (5, 8, 9) to maintain the phylogenetic relationship of D-J-C cluster 2 among all other mammalian species.

**Figure 4:**
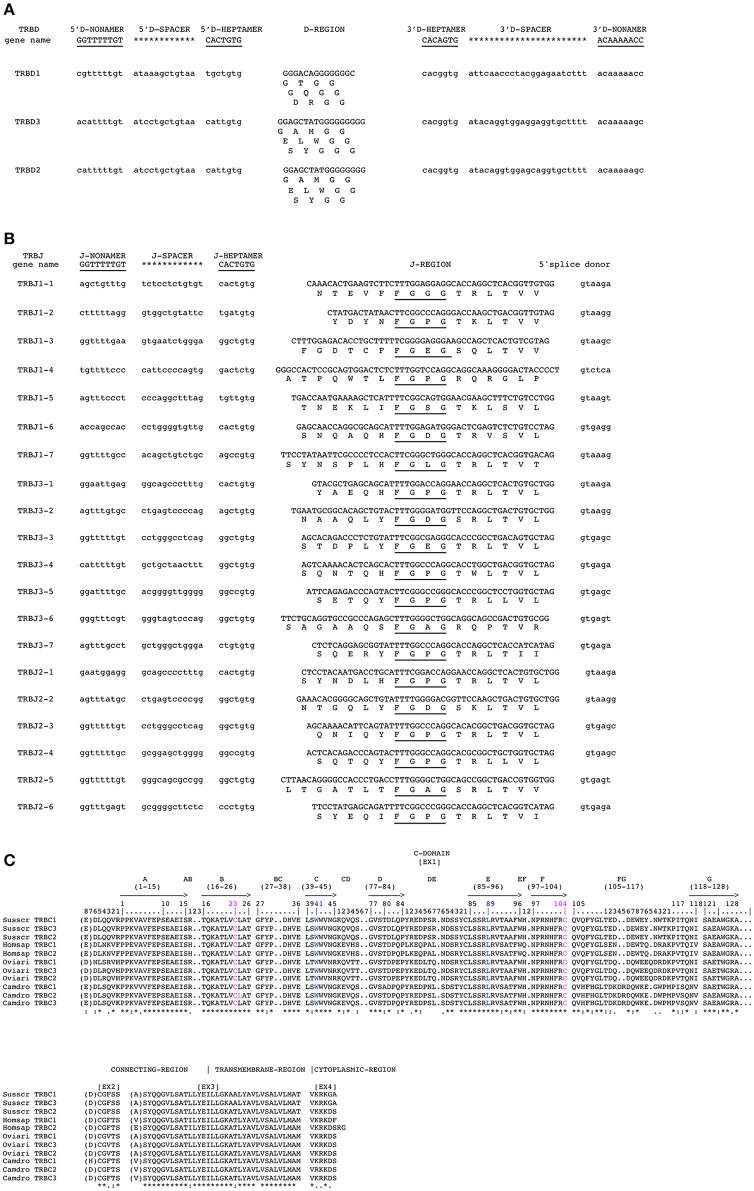
Nucleotide and deduced amino acid sequences of the pig TRBD **(A)**, TRBJ **(B)** and TRDC **(C)** genes. The consensus sequence of the heptamer and nonamer is provided at the top of the figure and is underlined. The numbering adopted for the gene classification is reported on the left of each gene. In **(A)**, the inferred amino acid sequence of the TRBD genes in the three coding frames are reported. In **(B)**, the donor splice site for each TRBJ is shown. The canonical FGXG amino acid motifs are underlined. In **(C)**, IMGT Protein display of the pig TRBC gene as derived from the alignment by Clustal W with the human, sheep and dromedary Cβ proteins. The descriptions of the strands and loops were collected according to the IMGT unique numbering for the C-DOMAIN (26).

The nucleotide and deduced amino acid sequences of the three TRBD genes are shown in Figure 4A. They consist of a 14 bp (TRBD1), 17 bp (TRBD3) and 16 bp (TRBD2) G-rich stretches that can be productively read through their three coding phases and encode 2**-**3 glycine residues, depending on the phase. The RSs that flank the 5′ and 3′ sides of the coding region are well conserved with respect to the consensus.

The nucleotides and deduced amino acid sequences of all the TRBJ genes identified in the region are reported in Figure 4B. They were classified, based on the international nomenclature [IMGT®, http://www.imgt.org, (16, 27)], according to the following TRBC gene and numbered in agreement with the genomic position within the respective cluster. All genes are typically 44**–**55 bp in length and conserve the canonical FGXG amino acid motif, whose presence define the functionality of the J genes. Each TRBJ is flanked by a 12 RS at the 5′ end and by a donor splice site at the 3′ end. All the RSs are well conserved with respect to the consensus except for the TRBJ1-4 heptamer where the G nucleotide located in the fifth nucleotide position is mutated to C.

Like those in all known mammalian species, the three pig TRBC genes are composed of four exons and three introns as widely described in Eguchi-Ogawa et al. (6). The pig TRBC genes encode a similar protein of 177 amino acids. The difference of nine nucleotides between them result in four amino acid (AA) changes, one in the transmembrane (TM) and three in cytoplasmic regions (Figure 4C). The C-domain encoded by EX1 is 129 AA in length as in humans because of the identical length of the FG loop with respect to other artiodactyl species. The C region also comprises a connecting region (CO) of 21 AA (encoded by EX2 and the 5′ part of EX3) with a cysteine involved in the interchain disulphide binding, a TM of 21 AA (encoded by the 3′ part of EX3 and the first codon of EX4) and a cytoplasmic region (CY) of 5 AA (encoded by EX4).

### Genomic structure and comparative analysis of the pig TRB region

To provide further information on the genomic architecture of the pig TRB locus, the determined sequence was first screened with the RepeatMasker program to analyse the compositional properties (G + C content) and to identify interspersed repeats (Supplementary Table S4). The GC content in pig was 46.41%, which is higher than that among all the mammalian TRB loci analyzed so far [see Supplementary Table S2 in (3)]. In pig, the density of total interspersed repeats is 38.57%. The most abundant repeat elements are SINEs (15.65%) and LINEs (14.62%); the percentage of SINEs in the pig locus is higher than that in the human and dog loci (15.65% vs. 6.62 and 7.19%, respectively), whereas the amount of LINEs, predominantly that of LINE 1, is similar in all species.

**Figure 5:**
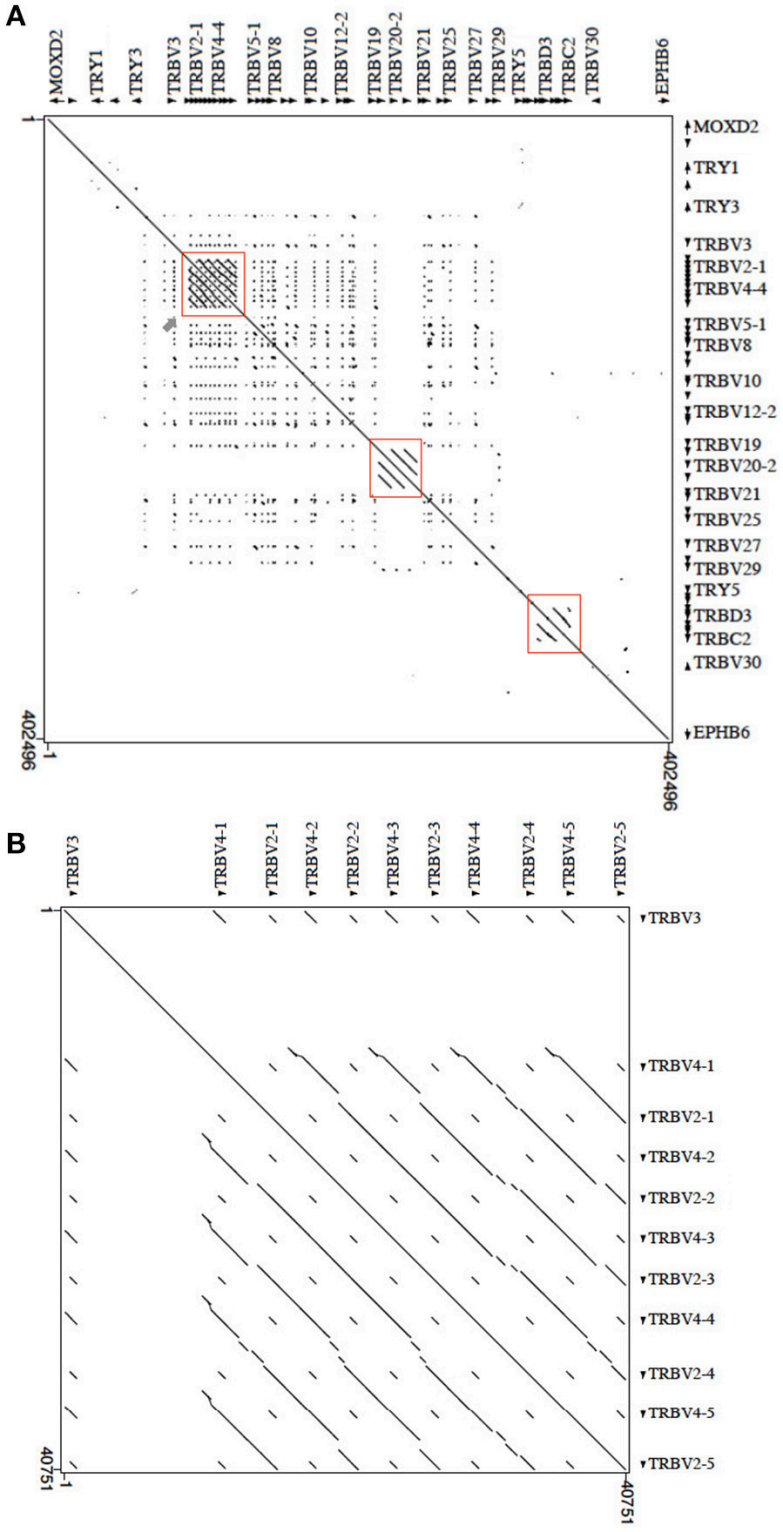
Dot-plot of the pig TRB locus sequence against itself. **(A)** With the exception of the main diagonal, the dots and diagonal lines indicate internal homology units in the sequence. The red boxes show the two TRBV regions underwent to duplication events and the internal homology of the D-J-C cluster portion. The gray arrow points to the homology region enlarged in **(B)**. **(B)** The pattern of parallel lines indicates the duplicated region of about 28 kb containing the TRBV2 and TRBV4 genes.

The masked pig sequence was then aligned with itself using the PipMaker program (17), and the alignment was expressed as a percentage identity plot (pip) (Supplementary Figure S1). The presence in the pip of superimposed lines indicates the occurrence of redundant matches along the entire region. The clearest matches correspond to all pig TRBV, except TRBV1 and TRBV30, due to the homology among genes. This is confirmed by the dot-plot matrix, where the high level of nucleotide identity between TRBV genes and between the D-J-C gene clusters is indicated by dots and diagonal lines (Figure 5). Inspection of the matrix allowed us to recognize, in addition to the perfect main diagonal line indicating the match of each base with itself, parallel lines identifying within the locus three duplicated regions. The first is approximately 28 kb in length, in which the TRBV2 and TRBV4 subgroup genes have arisen through a series of five tandem duplication events (Figure 5B). The homology unit is interrupted due to insertions or deletions, subsequent to the initial duplication of the whole region. Two lines parallel to the main diagonal identify the duplicated region of approximately 8 kb, where the TRBV20 gene subgroup lies. In this regard, the continuous homology lines (Figure 5A), the repetitive structure within the duplicated units as shown in the pip (Supplementary Figure S1), as well as the high nucleotide identity between the TRBV20 genes, highlight the occurrence of a recent duplication event. Finally, parallel lines characterize the internal homology of the D-J-C cluster duplications (Figure 5A).

Duplication events at the 5′ end of the TRB locus have also occurred in dog (3), with the TRBV2 and TRBV4 subgroup genes as well as TRBV3 involved in these events. Supplementary Figure S2 shows the sequence comparison dot-plot graph between the corresponding pig and dog TRBV portions (from MOXD2 to PRSS2). The matrix highlights a high level of nucleotide identity between the pig and dog TRB genes, as indicated by a nearly contiguous co-linearity between the two sequences (Supplementary Figure S2A). Parallel lines are evident at the 5′ end of the region, where the TRBV2, TRBV3, and TRBV4 subgroups lie. Homology units corresponding to TRBV4-TRBV2 gene duplications are evident (blue rectangles). In pig, a large LINE sequence (Supplementary Figure S1) separates the TRBV3 gene from the TRBV2 and TRBV4 genes, and it interrupts the parallel lines of homology either within the pig matrix (red rectangle in Figure 5B) and in the comparison with the dog counterpart (Supplementary Figure S2B).

Parallel lines in the dog TRBV20 gene region and homology units between the TRY genes are also present.

#### Evaluation of the TRB repertoire

The functional competency of the pig TRBV genes predicted in the genome assembly was first analyzed against the pig cDNA clone dataset available at IMGT/LIGM-DB (http://www.imgt.org). Comparison of the germline with the expressed sequences allowed us to ascertain that at least one cDNA perfectly matches the corresponding germline sequences for 11 TRBV genes (Table 1). Moreover, 15 germline TRBV genes showed a nucleotide identity from 97 to 99% to one cDNA. We referred to these as alleles based on the assumption that sequences sharing >97% of nucleotide identity represent the same gene. In this way, the functional competency of most TRBV genes was confirmed. As expected, no corresponding cDNAs were found for the nine germline TRBV genes classified as pseudogenes. However, two TRBV genes (TRBV5-3 and TRBV14), considered functional, seem to not have any corresponding cDNA. However, three genes identified as pseudogenes (TRBV5-2, TRBV6 and TRBV12-2) were found to have a corresponding cDNA. Although polymorphism conditions cannot be ruled out, we believe that sequencing errors within the genomic assembly may justify this discrepancy. In fact, an unfortunate side effect of the highly-contiguous PacBio sequencing is its high error rate (23).

**Table 1:** Correspondence between the germline and expressed TRBV genes.

**TRBV gene**	**Functionality**	**Corresponding cDNA clone** **accession number**	**Nucleotide** **identity %**
TRBV1	F	AY690918	99.7
TRBV2-1	P	–	–
TRBV2-2	P	–	–
TRBV2-3	P	–	–
TRBV2-4	F	AY691163	97.1
TRBV2-5	P	–	–
TRBV3	F	AY691035	97.9
TRBV4-1	F	AY691152	99.7
TRBV4-2	P	–	–
TRBV4-3/4	F	AY691156	100
TRBV4-5	F	AY691000	99.4
TRBV5-1	F	AY691018	99.7
TRBV5-2	P	AY691147	97.7
TRBV5-3	F	–	–
TRBV6	P	AY690936	99.1
TRBV7-1	F	AY691001	100
TRBV7-2	F	AY691161	100
TRBV8	P	–	–
TRBV10	F	AY690909	100
TRBV11	F	AY690947	99.1
TRBV12-1	F	AY691138	99.7
TRBV12-2	P	AY690854	99.7
TRBV14	F	–	–
TRBV15	F	AY691160	100
TRBV19	F	AY691020	99.4
TRBV20-1	F	AY690891	100
TRBV20-2	F	AY690903	100
TRBV20-3	F	AY690934	98.5
TRBV21	F	AY690915	100
TRBV22	P	–	–
TRBV23	P	–	–
TRBV24	F	AB079527	100
TRBV25	F	AY690938	99.4
TRBV27	F	AY690919	98.8
TRBV28	P	–	–
TRBV29	F	AY691002	100
TRBV30	F	AY690884	100

*The nucleotide identity and the Accession Number of each corresponding cDNA clone are reported. The functionality is referred to the germline genes*.

To evaluate the participation of germline TRBV genes in the generation of the adult pig TRB repertoire, we compared 197 clones (163 PBL and 34 in thymus) from Butler's cDNA collection (10) to the germline genes to determine the use of the TRBV and TRBJ genes.

**Figure 6:**
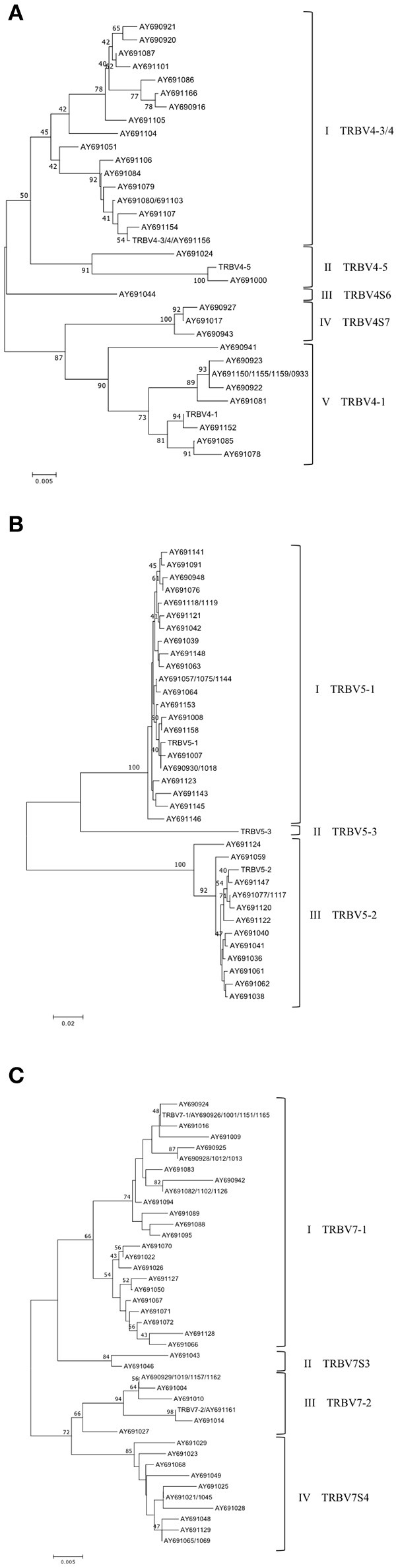
Evolutionary relationship of the TRBV4 **(A)**, TRBV5 **(B)** and TRBV7 **(C)** genes. The evolutionary history was inferred using the Neighbor-Joining method (21). The percentage of replicate trees in which the associated genes clustered together in the bootstrap test (100 replicates) are shown next to the branches (24). The trees are drawn to scale, with branch lengths in the same units as those of the evolutionary distances used to infer the phylogenetic trees. The evolutionary distances were computed using the p-distance method (22) and are in the units of the number of base differences per site. The analysis involved 33 (in **A**), 35 (in **B**) and 41 (in **C**) nucleotide sequences. Codon positions included were 1st+2nd+3rd+Noncoding. All positions containing gaps and missing data were eliminated. There were a total of 333 **(A,B)** and 348 **(C)** positions in the final dataset. The evolutionary analyses were conducted in MEGA7 (20).

Based on the percentage of nucleotide identity (as described above), the expressed TRBV genes have been unambiguous assigned to the corresponding germline TRBV genes for the one-member TRBV gene subgroups as well as for the TRBV2 and TRBV12 gene subgroups (data not shown). In contrast, the comparison has showed disparity between the germline and expressed TRBV genes for the TRBV4, TRBV5, and TRBV7 multi-member gene subgroups. In fact, the percentage of nucleotide identity alone did not allow us to determine if the TRBV genes, within the high number of cDNAs, represented allelic variants of previously identified genes or products of additional genes absent in the current assembly. Therefore, we conducted a phylogenetic analysis of these TRBV subgroups. The nucleotide sequences (from L-PART1 to FR3-IMGT) of all TRBV4, TRBV5, and TRBV7 expressed genes were combined with the corresponding germline sequences in the same alignment and an unrooted phylogenetic tree was made for each TRBV subgroup using the NJ method (Figure 6). The TRBV4 tree resolves the sequences into five groups (Figure 6A). The first and the fifth groups contained 18 and 11 cDNAs together with the germline TRBV4-3/4 and TRBV4-1 genes respectively, allowing us to classify these TRBV4 cDNAs. Two cDNAs and the germline TRBV4-5 gene comprise the second group. In the third and fourth groups, there are no corresponding genomic sequences; therefore, they could represent new genes provisional classified as TRBV4S6 and TRBV4S7, respectively.

In the TRBV5 tree (Figure 6B), 38 cDNA sequences were grouped into two clusters containing the functional germline TRBV5-1 and the possibly functional TRBV5-2 gene. No cDNAs were clustered with the TRBV5-3 gene.

Finally, the phylogenetic tree for the TRBV7 subgroup, generated by the alignment of 53 cDNAs with functional germline TRBV7-1 and TRBV7-2, resolves the TRBV7 sequences into 4 groups (Figure 6C). The first and third groups contains 30 and 9 cDNAs together with the germline TRBV7-1 and TRBV7-2 sequences, respectively. The second (two clones) and fourth (12 clones) groups do not have corresponding genomic genes, so they may represent new genes. The provisional nomenclature TRBV7S3 and TRBV7S4 has been attributed to these supposed new TRBV7 genes.

A phylogenetic tree was also constructed for the TRBV20 gene subgroup, given that the TRBV20-2 gene exhibits a nucleotide identity >97% with the other two germline TRBV20 genes, which makes it impossible to assign a cDNA to its genomic sequence. Therefore, the TRBV20 cDNA clones were aligned with the functional genomic sequences and the phylogenetic tree produced three groups that allowed us to classify the TRBV20 cDNAs as TRBV20-1 and TRBV20-3 (Supplementary Figure S3).

**Figure 7:**
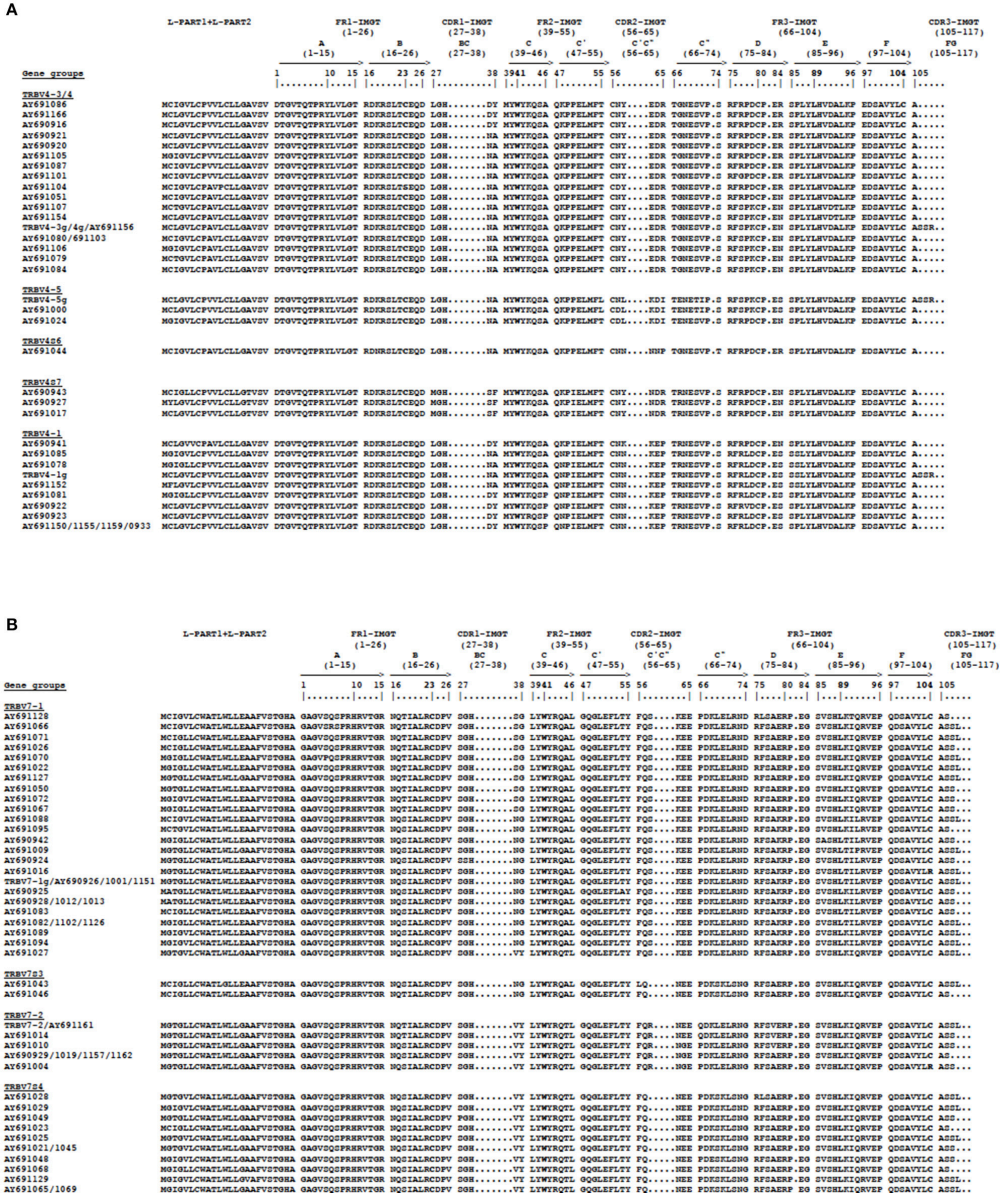
The IMGT Protein display of the pig TRBV4 **(A)** and TRBV7 **(B)** germline and cDNA genes. The gene groups represent the phylogenetic clusters of Figure 6. The TRBV cDNA genes are indicated by the Accession number.The description of the strands and loops and of the FR-IMGT and CDR-IMGT is according to the IMGT unique numbering for the V-REGION (25). The five conserved amino acids of the V-DOMAIN (1st-CYS 23, except for the TRBV6 pseudogene, CONSERVED-TRP 41, hydrophobic AA 89, 2nd-CYS 104 and J-PHE 118) are indicated in bold.

To corroborate the phylogenetic results that led to the identification of new possible TRBV4 and TRBV7 genes missing in the genome assembly, we analyzed the structure of these genes. Hence, the deduced amino acid sequences of the cDNA and germline TRBV4 and TRBV7 genes were manually aligned according to the unique IMGT numbering to maximize homology and were grouped according to the phylogenetic clustering (Figure 7). Alignment of the TRBV4 amino acid sequences showed that they exhibit a similar length at all intervals, while they vary in their amino acid composition (Figure 7A). A careful analysis of the variations among the groups reveals that the amino acid changes are in specific locations; if the amino acid composition of CDR1-IMGT and CDR2-IMGT is substantially preserved within each group, the last three amino acids of the CDR2-IMGT (pos. 63–65) would be most predictive of TRBV4 gene identity. In contrast, the amino acid alignment of the TRBV7 sequences shows that the more consistent difference among groups lies in the CDR2-IMGT length, with the TRBV7S3 and TRBV7S4 groups having one amino acid less in the CDR2-IMGT with respect to TRBV7-1 and TRBV7-2, respectively (Figure 7B). However, in this case, the amino acid composition of the CDR1 and CDR2 loops is markedly preserved within each group.

The entire expression profile of the pig TRB genes in PBL and thymus is summarized in Table 2 and Table 3. Overall, the expression of 26 TRBV genes belonging to 16 subgroups was detected. The most common TRBV gene used in PBL was the TRBV12-1, represented in 37 different transcripts, followed by TRBV7-1 and TRBV5-1 with 25 and 23 cDNA clones, respectively (Table 2). One additional finding is the absence in PBL of TRBV genes expressed in thymus as TRBV1, TRBV6, TRBV11, TRBV20, TRBV25, and TRBV27 (Table 3). No prevalent expression of particular TRBV genes was noted in thymus. A total of 18 different TRBJ sequences were also detected. All TRBJ genes were expressed, except TRBJ1-4 and TRBJ3-6. In fact, TRBJ1-4 was previously classified as a pseudogene (Figure 4B), whereas the TRBJ3-6, even if seems to be functional, has never been found to be expressed, as confirmed by a previous study (6). The TRBJ genes most commonly used are the TRBJ1-2 and the TRBJ2-6 in PBL (Table 2). The TRBJ gene was not been recognized in only three clones because of nucleotide trimming.

**Table 2:** The TRBV and TRBJ genes repertoire of the peripheral blood lymphocytes.

**TRBV gene**	**TRBJ cluster**																			
	**TRBJ1**						**TRBJ3**						**TRBJ2**						**nd**	**Total**
	**1-1**	**1-2**	**1-3**	**1-5**	**1-6**	**1-7**	**3-1**	**3-2**	**3-3**	**3-4**	**3-5**	**3-7**	**2-1**	**2-2**	**2-3**	**2-4**	**2-5**	**2-6**	
V2-4									1									1		2
V3	1																			1
V4-1		1				1		1			1							3		7
V4-3/4	1	3	1	3			1		2	1				1		1		1		15
V4-5																		1		1
V4S6		1																		1
V4S7		1																		1
V5-1		4		2	1	3	1			1	1	1	1	1	1	1	2	2	1	23
V5-2	1				1		2	1			1		1	1	1	1		1	1	12
V5S4		1																		1
V7-1	2	2	3	1	1	1	1	1	1	2	2				1	1	2	4		25
V7-2				1					2				1		1		1	2		8
V7S3		5	1			1		1						1	1		1	1		12
V7S4	1		1																	2
V12-1	2	5	3	1			1	2	1	2	5				4		3	7	1	37
V15	1	1							1							1		1		5
V19											1									1
V21	1	3	1	1		1							1							8
V29										1										1
Total	10	27	10	9	3	7	6	6	8	7	11	1	4	4	9	5	9	24	3	163

nd: the TRBJ gene is not detectable

**Table 3:** The TRBV and TRBJ genes repertoire of the thymus lymphocytes.

**TRBV gene**	**TRBJ cluster**												
	**TRBJ1**				**TRBJ3**			**TRBJ2**					**Total**
	**1-1**	**1-2**	**1-3**	**1-7**	**3-3**	**3-4**	**3-5**	**2-1**	**2-2**	**2-3**	**2-4**	**2-6**
V1						1							1
V4-1		1		1			1			1			4
V4-3/4	1	2											3
V4-5				1									1
V4S7			1					1					2
V5-1					1				1				2
V6		1											1
V7-1		2		1			1				1		5
V7-2		1											1
V11	1												1
V20-1											1		1
V20-3	2			1								1	4
V12-1					1			1	1				3
V21							1						1
V25			1				1		1				3
V27							1						1
Total	4	7	2	4	2	1	5	2	3	1	2	1	34

#### Analysis of the D-J-C rearrangements

For the determination of the TRBD genes and a close inspection of the CDR3-IMGT, the nucleotide sequences of all expressed products (197 cDNA clones), from codon 105 to 117 according to the unique numbering (25), have been excised and analyzed in detail (Supplementary Table S5). The deduced amino acid sequences of the CDR3 loop reveal that it is heterogeneous in regard to amino acid composition and length without specific differences in relation to TRBV or TRBJ gene usage. The mean length of the CDR3 loop was 12.7 amino acids (range 9–18 amino acids) for thymus and 12.2 amino acids (range 5–18) for PBL.

In comparison with genomic TRBD, the sequences located in the CDR3 regions were considered to belong to a TRBD gene if they constituted a stretch of at least five consecutive nucleotides. In this way, the TRBD gene was unambiguously identified in 18 out of 34 sequences in thymus (59.2%), with TRBD1 present in 16 clones (47.0%) and TRBD3 in two clones. The remaining 16 sequences either do not have an identifiable TRBD gene (12 clones), or it is not possible to distinguish between TRBD3 and TRBD2 (three clones) or between TRBD1, TRBD3, and TRBD2 (one clone) because of their similar germline sequences (Supplementary Table S5). Likewise, in PBL, TRBD was unambiguously identified in 88 out of 163 sequences (54%) with TRBD1 present in 82 clones (50.3%) and TRBD3 in six clones. The remaining 75 sequences either do not have an identifiable TRBD gene (51 clones), or it is not possible to distinguish between TRBD3 and TRBD2 (19 clones) or between TRBD1, TRBD3 and TRBD2 (5 clones).

The absence of a TRBD region could be interpreted as the presence of a direct V-J junction. However, it is also possible that nucleotide trimming masked the initial participation of the TRBD gene during rearrangement. In four cases (AY691005, AY691023, AY691073, and AY691083), two TRBD genes in the CDR3 region are present, while a large number of cDNA clones present within the D region contain nucleotide substitutions with respect to the germline sequences.

Because the genomic organization of the D-J-C region is known, a formal interpretation of the DJC arrangement was also possible. The intra-cluster rearrangements are clearly appreciable in 49 clones with 48 TRBD1-TRBJ1 and one TRBD3-TRBJ3 recombination. Fifty-five rearrangements can be interpreted by direct 5′ to 3′ joining across the clusters (inter-cluster rearrangements), with 23 TRBD1-TRBJ3, 26 TRBD1-TRBJ2 and 6 TRBD3-TRBJ2 recombinations. Interestingly, we also observed six TRBD3 or TRBD2-TRBJ1 joinings (AY690929, AY690933, AY690937, AY691046, AY691134, AY691166). Because D-J-C cluster 3 and D-J-C cluster 2 are located downstream of D-J-C cluster 1 within the TRB locus, this junction may be explained by chromosomal inversion or by trans-rearrangement with a greater probability, which occurs during TRB locus recombination.

## Discussion

The TRB locus has been extensively studied in many different mammalian species. However, until now, fragmented information has been available on the genomic organization of the pig TRB locus.

The present study, updates and completes the genomic organization of the pig TRB locus based on the sequence of the whole genome assembly Sscrofa11.1. One striking finding is the annotation of 38 TRBV genes grouped into 24 subgroups that span approximately 300kb (Figure 1 and Supplementary Table S1). The functional germline repertoire seems partially reduced, considering that 12 out of 38 (31.5%) TRBV genes are classified as pseudogenes (Supplementary Table S3), in contrast to those in human (19%) (IMGT Repertoire, http://www.imgt.org) and rabbit (22%) (4). However, a higher number of pseudogenes are present in the dog genome (43%) (3). As in other artiodactyl species, the pool of TRBV genes is located upstream of three in tandem TRBD-J-C clusters, followed by a TRBV gene with an inverted transcriptional orientation and distributed in a further 100 kb (Figure 1). The MODX2 and EPHB6 genes flank the 5′ and 3′ ends of the TRB locus, respectively, while four TRY are intercalated within the TRB genes; three of them are located downstream of the TRBV1 gene, and one lies between the last TRBV gene and the first TRBD-J-C cluster.

The genomic data support the concept that the number of TRBV genes is the parameter that varies mostly among species, with some species having more than 100 TRBV genes, while other species have less than 40. In cattle, 134 TRBV genes distributed in 24 subgroups have been identified; the TRBV genes present in the human, rabbit, dog, dromedary and pig genome number 68 in 31 subgroups, 74 in 24 subgroups, 37 in 25 subgroups, 33 in 26 subgroups and 38 in 24 subgroups, respectively [IMGT Repertoire, http://www.imgt.org; (3, 4, 7–9), and this study]. Evidently, variations also occur among species within the same mammalian order, suggesting that evolutionary pressures in each species tend to determine the total number of TRBV genes. However, phylogenetic clustering of the TRBV genes (Figure 2) reveals that the birth of distinct TRBV subgroups occurred prior to the radiation of mammals. Therefore, gene duplication within each subgroup, rather than the emergence of different gene subgroups, is the major mode of evolution of TRBV genes in various species, including pig.

Nevertheless, in some cases, the evolutionary dynamics seem to be shared among species. In pig, the more consistent duplications are located at the 5′ end of the TRB locus, involving the TRBV2 and TRBV4 subgroups, which are incorporated into four homology units (Figure 5). These duplications appear similar to the most remarkable duplications that characterize the dog TRB locus, where, in addition to TRBV2 and TRBV4, the TRBV3 gene subgroup is also involved (Supplementary Figure S2). Hence, the evolutionary event might have been the same in pigs and dogs, given the close phylogenetic relationship between artiodactyls and carnivores. Alternatively, the duplication events might have occurred independently in the two species but involved the same genes as a result of the fitness requirement. Similarly, a recurrent pattern of duplications composed of the TRBV5, TRBV6, and TRBV7 subgroups has occurred in the human and rabbit TRB loci [IMGT Repertoire, http://www.imgt.org; (4)].

Notably, rare duplications have occurred at the 3′ end of the TRB locus, but in this case, single subgroups are involved, such as TRBV20 and TRBV21 in cattle (7), TRBV21 in rabbit (4) and TRBV20 in pig (Figure 5). The duplication events seem to be constantly evolving, as is evident from the nucleotide identity among member genes of some subgroups ranging from more than 97% (TRBV20 genes) to 100% (TRBV4 genes).

The genome analysis revealed that repetitive sequences such as SINEs and LINEs could have contributed to the gene duplications and the resulting locus morphology (Supplementary Figure S1 and Supplementary Table S4). In contrast to human and dog but similarly to rabbit, LINEs (14.62%) and SINEs (15.65%) are the most abundant repetitive sequences in the pig TRB locus. For instance, SINEs and LINEs are most abundant in the homology unit in which the TRBV20 genes lie, and they could have been a substrate for the duplication event.

In contrast to the TRBV genes, the pig TRBC genes, as in the other species (5, 8, 9), encode very similar products (Figure 4C), indicating the existence of an intra-species homogenization process due to the strong functional constraint of the constant portions of the TR chains.

Present knowledge of the genomic organization of the pig TRB locus has allowed us to elucidate the participation of the germline TRBV genes in the adult expressed repertoire. A cDNA collection of 197 independent clones derived from adult thymus and PBL were retrieved (10), and the members of 16 out of 19 functional germline TRBV gene subgroups were identified, including the TRBV6, which was classified as a pseudogene in the assembly (Table 1).

We found a biased usage pattern of the TRBV genes in the PBL (Table 2). A high relative level was found for TRBV12-1 (22.6%); those of TRBV7-1 and TRBV5-1 were at moderate frequencies (15.3 and 14.1%, respectively), while other TRBV genes were used relatively less. The TRBV5-2 and TRBV12-2 genes, classified as pseudogenes, were also found to be expressed. No prevalent expression of particular TRBV genes was found in thymus (Table 3), even if the number of analyzed clones was reduced. Overall, the expressed repertoire seems more broad than the germline repertoire. We ascribe this result to a high grade of polymorphism because the cDNA clones were derived from diverse animals (10), although sequencing errors within the genomic assembly may also justify this discrepancy. Moreover, one interesting finding is the identification in the PBL cDNA clones of four TRBV genes missing in the assembly, belonging to the TRBV4 and TRBV7 multi-member gene subgroups (Table 2 and Figure 6). Although it cannot be excluded that a substantial number of genes, predominantly from the large subgroups, are still absent from the sequenced pig genome, we suppose, more likely, the possible existence of insertion/deletion-related polymorphisms (IDRPs), which can lead to intra-species variation in the genomic TRBV gene repertoire, as described in human for the same TRBV gene subgroups (28).

The amino acid sequence comparison of the new TRBV genes among each subgroup showed that the structural composition and organization of the CDR2-IMGT is predictive of TRBV4 and TRBV7 gene identities (Figure 7). The current dogma regarding TR recognition of the pMH proposes that the germline-encoded CDR1β and CDR2β loops mainly contact the polymorphic region of the MH surface, while the somatically rearranged hypervariable CDR3β loop principally recognizes the peptide bound in the MH groove. Therefore, the diversity observed in the CDR2-IMGT of the TRBV4 and TRBV7 gene repertoire can be related to the hyperpolymorphism of the pig MH molecules (29).

Furthermore, the presence of a consistent number of cDNA clones allows us to perform a comprehensive analysis of the CDR3 region and its relationship with pig TRB genomic organization (Supplementary Table S5). The CDR3 region contained a heterogeneous amino acid composition, and its mean length was approximately the same in thymus and peripheral blood with respect to human and dog, indicating that the CDR3 length in the β chain is essential for T cell function. Our analyses also suggest an alternate TRBD usage; therefore, TRBD1 can account for approximately 50% of the total clones in both tissues (Supplementary Table S5). This functionality would reside in the greater efficiency of PDβ1 promoter activity (30). A striking conservation of PDβ1 and PDβ2 (as well as the PDβ3 promoter) among pig, sheep, human and mouse supports this observation (5).

Because of their similar nucleotide sequences, the relative usage of the TRBD2 and TRBD3 genes could not be identified (Supplementary Table S5).

The prominent utilization of the members of the TRBJ1 (especially TRBJ1-2) and TRBJ2 (particularly TRBJ2-6) sets with respect to the TRBJ3 set, as deduced from the cDNA collection, is a result of intra- and inter-cluster rearrangement, even if a small portion also arose from trans rearrangements as in sheep (31).

Overall, the data presented here show that the mechanisms that generate diversity in the pig β chain appear to adhere to the paradigms established in sheep, human and dog.

This study represent a base for future studies on pig adaptive immunity and could be especially relevant in understanding the TRBV expressed repertoire during porcine diseases, in swine models of human disease and in xenotransplantation.

## Author contributions

RA, SC, and SM designed research and wrote the paper. MB contributed to the genomic analysis. RA and SM contributed to the genomic and cDNA analysis. All authors have read and approved the final manuscript.

### Conflict of interest statement

The authors declare that the research was conducted in the absence of any commercial or financial relationships that could be construed as a potential conflict of interest.

## Supplementary material

Supplementary Figure S1 Pip of the pig sequence versus itself. The position and orientation of all the genes and repetitive sequences are indicated. The horizontal lines represent the ungapped alignments at the percentage similarity corresponding to the scale on the right.

Supplementary Figure S2 Dot-plot of pig/dog TRB genomic comparison. **(A)** Using the Pip-Maker program the pig TRBV region (fron MOXD2 to PRSS2, horizontal axis) has been plotted against the dog counterpart (vertical axes). The red boxes show the TRBV region underwent to duplication events. The gray arrow points to the homology region enlarged in **(B)**. **(B)** The pattern of parallel lines indicates the duplicated region in pig and dog containing the TRBV2, TRBV4 as well as the TRBV3 genes. The red box indicate a gap between the sequences caused by a pig LINE; whereas, the blue boxes indicate the TRBV4-TRBV2 homology units in the dog sequence (3).

Supplementary Figure S3 The NJ tree inferred from the pig TRBV20 gene sequences. The evolutionary analysis was conducted in MEGA7 (20). The optimal tree with the sum of branch length = 0.07454819 is shown. The percentage of replicate trees in which the associated taxa clustered together in the bootstrap test (1,000 replicates) is shown next to the branches (24). The tree is drawn to scale with branch lengths in the same units as those of the evolutionary distances used to infer phylogenetic trees. The evolutionary distances were computed using the p-distance method (22) and are in the units of the number of base differences per site. The analysis involved 8 nucleotide sequences. Codon positions included were 1st+2nd+3rd+Noncoding. All positions containing gaps and missing data were eliminated. There were a total of 332 positions in the final dataset.

Supplementary Table S1 Description of the TRB genes in the *Sus scrofa* chromosome 18 genome assembly (NCBI Reference Sequence NC_010460). The position of all genes and their classification and functionality are reported.

Supplementary Table S2 Description of the unrelated TRB genes in the *Sus scrofa* chromosome 18 genome assembly (NCBI Reference Sequence NC_010460). The position of all genes and their classification and functionality are reported.

Supplementary Table S3 Description of the *Susscr* TRBV pseudogenes.

Supplementary Table S4 Output file of the program RepeatMasker. Summary of the repeat content of pig TRB locus is shown. The GC levels are also indicated.

Supplementary Table S5 CDR3 nucleotide and predicted amino acid sequences retrieved from the TRB cDNA clones. CDR3-IMGT sequences are shown from codon 105 (the codon after the 2nd-CYS 104 of the V-REGION) to codon 117 (the codon before J-PHE 118 of the J-REGION) according to the unique numbering (25). The CDR3 nucleotide/amino acid sequence, and the classification of the TRBV, TRBD and TRBJ genes of each clone are also listed. Nucleotides of the 3'V-REGION and of the 5'J-REGION are indicated in uppercase letters. The sequences considered to present recognizable TRBD genes are indicated in bold lowercase letters and the nucleotide substitutions or insertions are underlined. Nucleotides that cannot be attributed to any V, D or J region (N-nucleotides), are indicated in lower cases on the left and on the right sides of the TRBD regions. The Accession number of the clones is reported.
