# Supplementary figures and images for: Overview of the Germline and Expressed Repertoires of the TRB Genes in Sus scrofa
Source: Front Immunol. 2018 Nov 5;9:2526. doi: 10.3389/fimmu.2018.02526 (PMC6230588; doi:10.3389/fimmu.2018.02526)

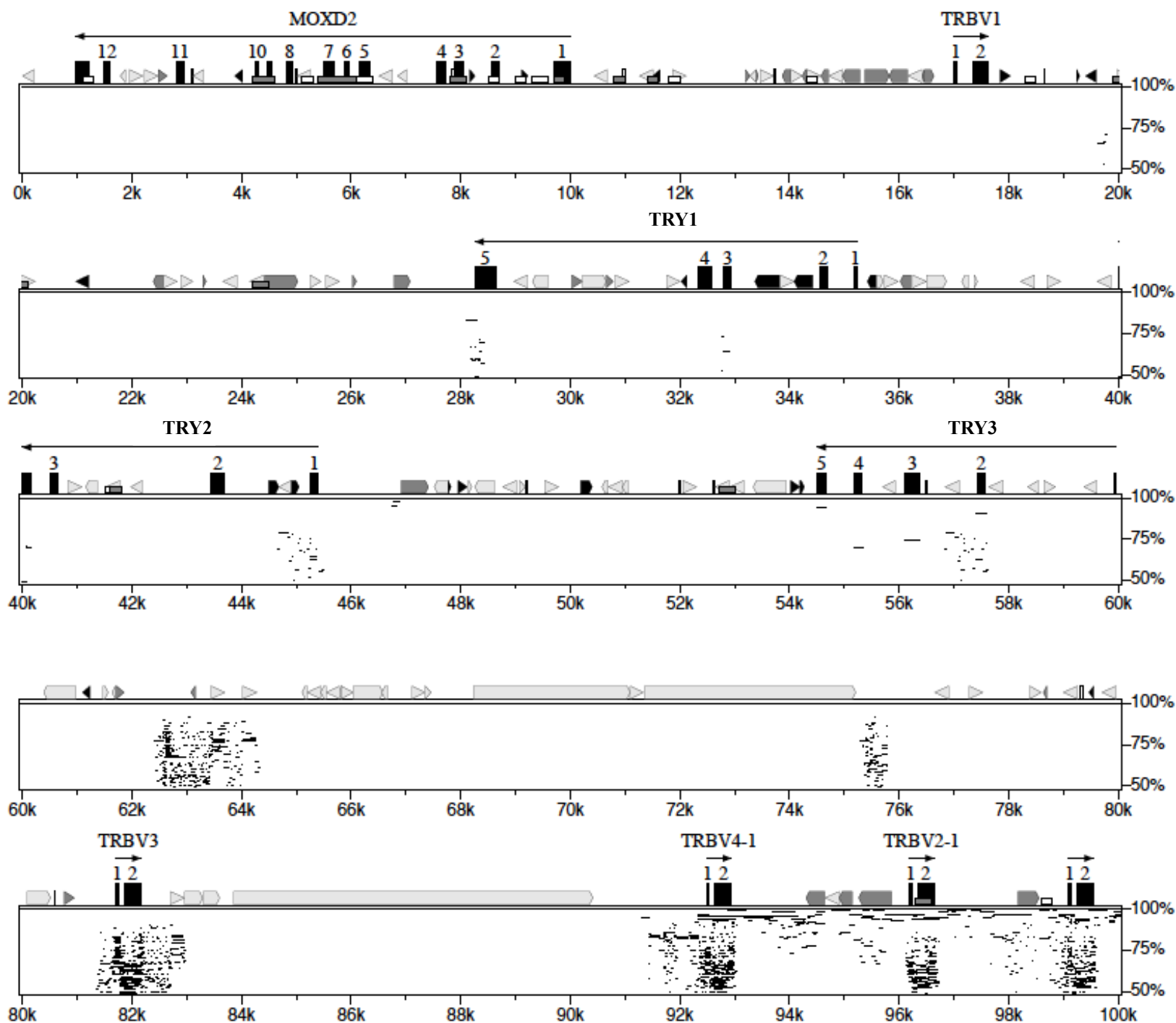

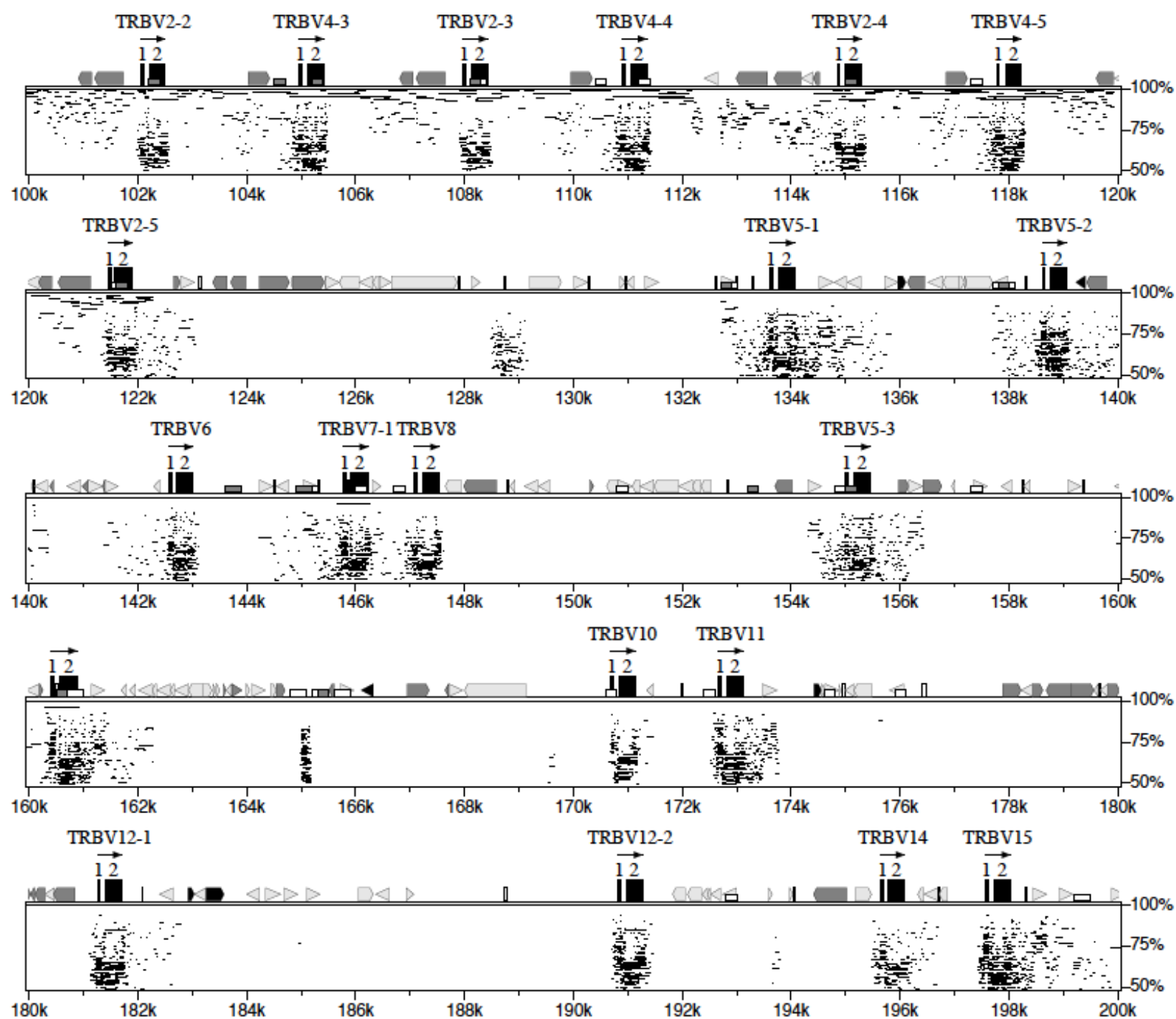

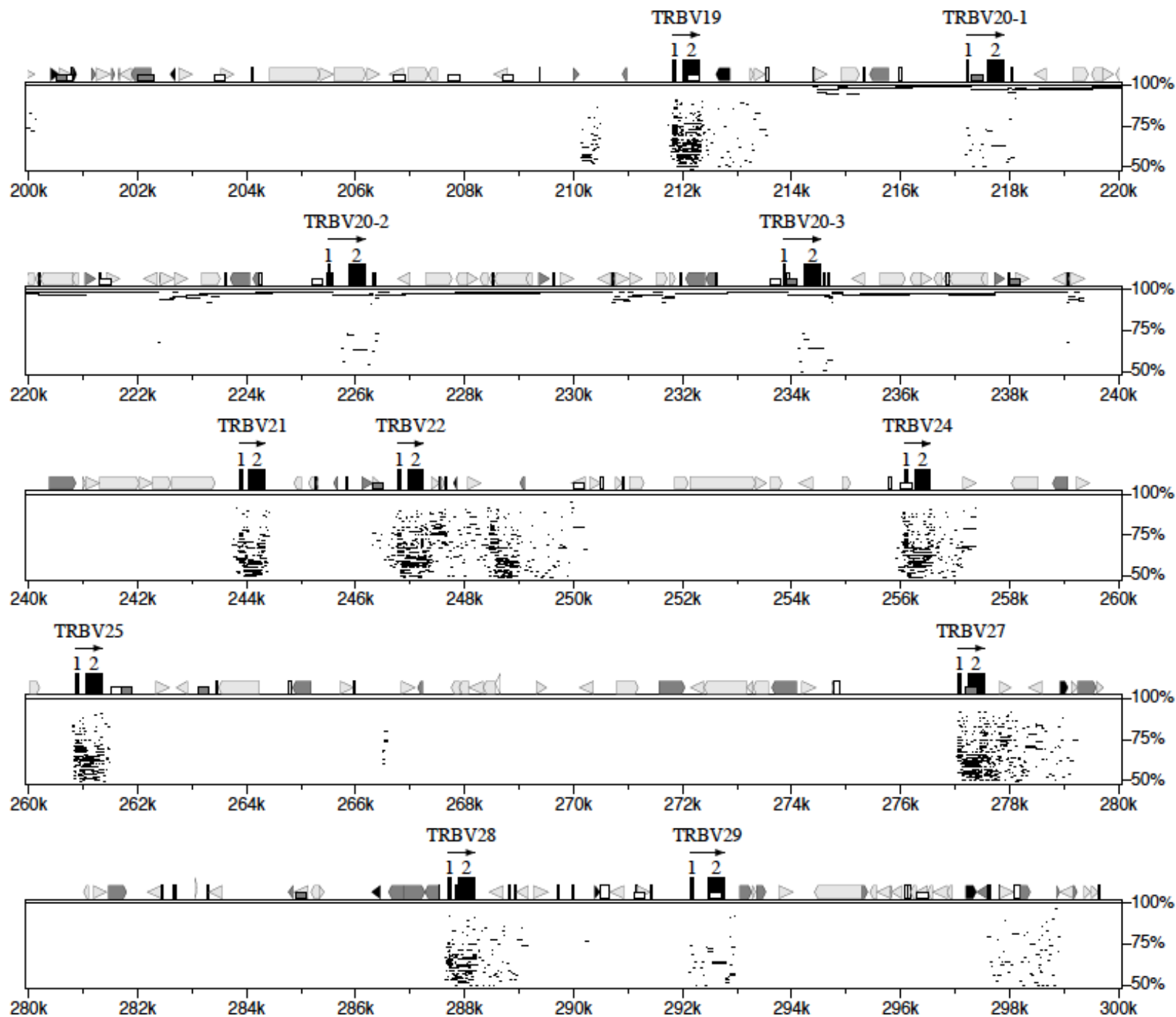

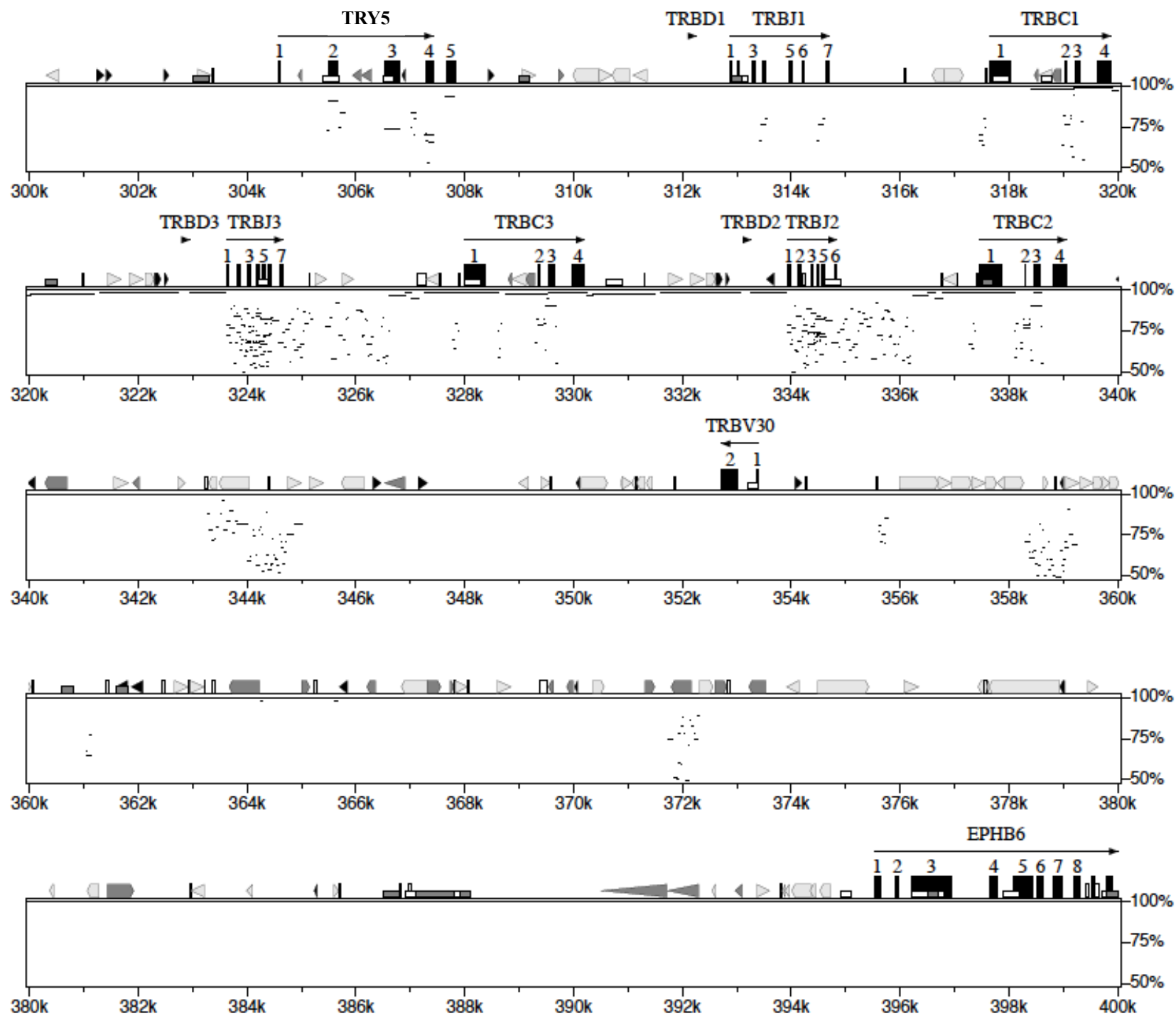

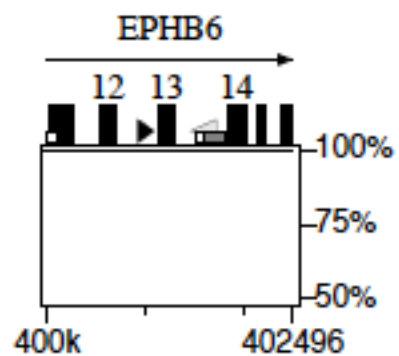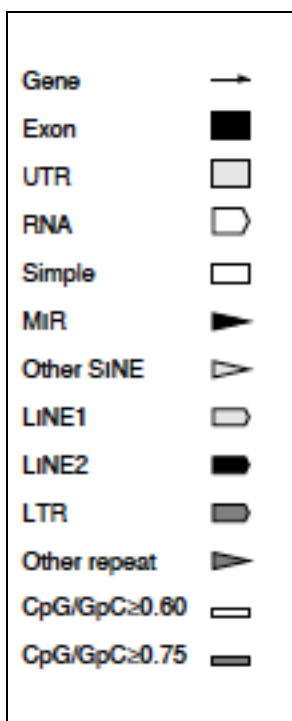

Supplement: Supplementary Figure S1 — Pip of the pig sequence versus itself. The position and orientation of all the genes and repetitive sequences are indicated. The horizontal lines represent the ungapped alignments at the percentage similarity corresponding to the scale on the right. [file Image_1.pdf]

Pig

a

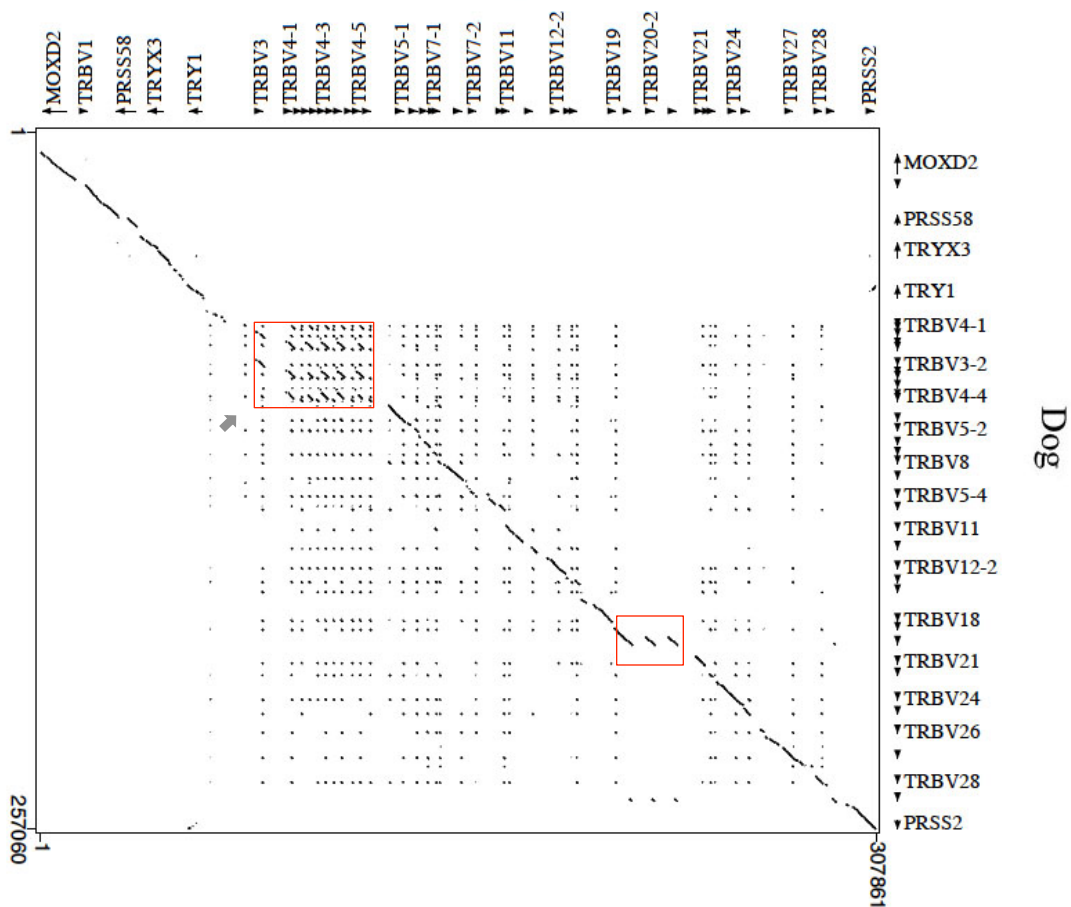

Pig

b

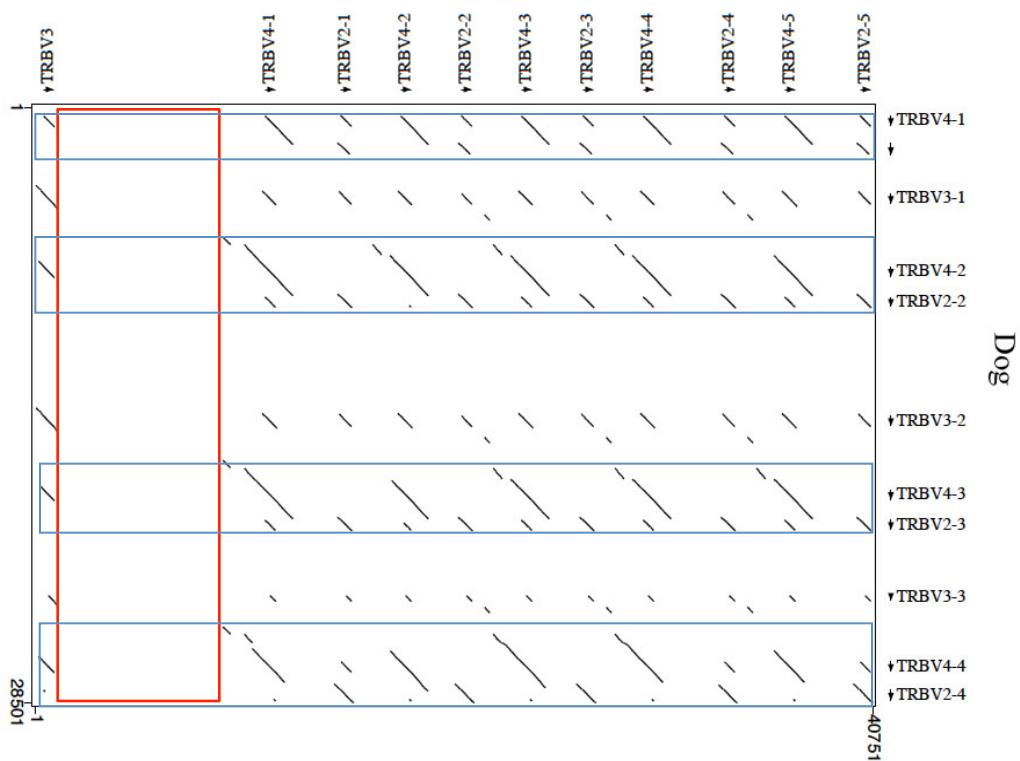

Supplement: Supplementary Figure S2 — Dot-plot of pig/dog TRB genomic comparison. (A) Using the Pip-Maker program the pig TRBV region (fron MOXD2 to PRSS2, horizontal axis) has been plotted against the dog counterpart (vertical axes). The red boxes show the TRBV region underwent to duplication events. The gray arrow points to the homology region enlarged in (B). (B) The pattern of parallel lines indicates the duplicated region in pig and dog containing the TRBV2, TRBV4 as well as the TRBV3 genes. The red box indicate a gap between the sequences caused by a pig LINE; whereas, the blue boxes indicate the TRBV4-TRBV2 homology units in the dog sequence (3). [file Image_2.PDF]

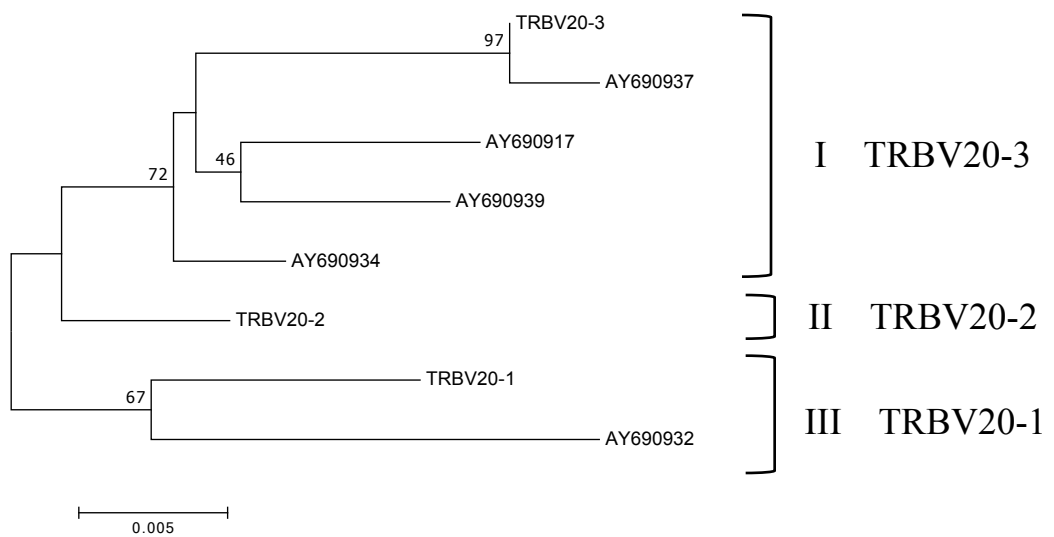

Supplement: Supplementary Figure S3 — The NJ tree inferred from the pig TRBV20 gene sequences. The evolutionary analysis was conducted in MEGA7 (20). The optimal tree with the sum of branch length = 0.07454819 is shown. The percentage of replicate trees in which the associated taxa clustered together in the bootstrap test (1,000 replicates) is shown next to the branches (24). The tree is drawn to scale with branch lengths in the same units as those of the evolutionary distances used to infer phylogenetic trees. The evolutionary distances were computed using the p-distance method (22) and are in the units of the number of base differences per site. The analysis involved 8 nucleotide sequences. Codon positions included were 1st+2nd+3rd+Noncoding. All positions containing gaps and missing data were eliminated. There were a total of 332 positions in the final dataset. [file Image_3.PDF]
