# Supplementary material for: Overview of the Germline and Expressed Repertoires of the TRB Genes in Sus scrofa
Source: Front Immunol. 2018 Nov 5;9:2526. doi: 10.3389/fimmu.2018.02526 (PMC6230588; doi:10.3389/fimmu.2018.02526)
Supplement: Supplementary Table S1 — Description of the TRB genes in the Sus scrofa chromosome 18 genome assembly (NCBI Reference Sequence NC_010460). The position of all genes and their classification and functionality are reported. [file Table_1.PDF]

Supplementary Table S1. Description of the TRB genes in the *Sus scrofa* chromosome 18 genome assembly (NCBI Reference Sequence NC_010460). The position of all genes and their classification and functionality are reported.

**Gene classification**	**Functionality**[b]	**Position**[a] (complement)
TRBV1	F	7734192-7733565
TRBV3	F	7669490-7669041
TRBV4-1	F	7658715-7658261
TRBV2-1	P	7655012-7642779
TRBV4-2	P	7652111-7651662
TRBV2-2	P	7649143-7642779
TRBV4-3	F	7640310-7639856
TRBV2-3	P	7643228-7642779
TRBV4-4	F	7640310-7639856
TRBV2-4	F	7636353-7629295
TRBV4-5	F	7633424-7632970
TRBV2-5	P	7629744-7629295
TRBV5-1	F	7617595-7617140
TRBV5-2	P	7612584-7612152
TRBV6	P	7608622-7608194
TRBV7-1	F	7605421-7604971
TRBV8	P	7604130-7603671
TRBV5-3	F	7596206-7595754
TRBV7-2	F	7590792-7590318
TRBV10	F	7580532-7580066
TRBV11	F	7578532-7578085
TRBV12-1	F	7569942-7569498
TRBV12-2	P	7560379-7559937
TRBV14	F	7555547-7555118
TRBV15	F	7553638-7553174
TRBV19	F	7539369-7538893
TRBV20-1	F	7533974-7533275
TRBV20-2	F	7525713-7525014
TRBV20-3	F	7517351-7516652
TRBV21	F	7507323-7506866
TRBV22	P	7504424-7503959
TRBV23	P	7502722-7502260
TRBV24	F	7495123-7494651
TRBV25	F	7490331-7489856
TRBV27	F	7474142-7473643
TRBV28	P	7463497-7462998
TRBV29	F	7459048-7458439
TRBD1	F	7438960-7438908
TRBJ1-1	F	7438336-7438289
TRBJ1-2	F	7438201-7438158
TRBJ1-3	F	7437920-7437871
TRBJ1-4	P	7437735-7437681
TRBJ1-5	F	7437248-7437198
TRBJ1-6	F	7437006-7436957
TRBJ1-7	F	7436550-7436498
TRBC1	F	7413713-7431331
TRBD3	F	7428265-7428210
TRBJ3-1	F	7427583-7427537
TRBJ3-2	F	7427390-7427340
TRBJ3-3	F	7427182-7427134
TRBJ3-4	F	7427032-7426984

TRBJ3-5	F	7426912-7426865
TRBJ3-6	F?	7426814-7426763
TRBJ3-7	F	7426589-7426543
TRBC3	F	7413713-7420998
TRBD2	F	7417949-7417895
TRBJ2-1	F	7417268-7417219
TRBJ2-2	F	7417068-7417018
TRBJ2-3	F	7416845-7416798
TRBJ2-4	F	7416731-7416684
TRBJ2-5	F	7416633-7416581
TRBJ2-6	F	7416407-7416361
TRBC2	F	7413713-7412150
TRBV30	F	7397804-7398474

[a] L-PART1/ V-exon for TRBV genes

[b] nd: not defined (indicates that the nt sequence of tfhe gene is incomplete and its functionality cannot be defined)
