# Supplementary material for: Overview of the Germline and Expressed Repertoires of the TRB Genes in Sus scrofa
Source: Front Immunol. 2018 Nov 5;9:2526. doi: 10.3389/fimmu.2018.02526 (PMC6230588; doi:10.3389/fimmu.2018.02526)
Supplement: Supplementary Table S2 — Description of the unrelated TRB genes in the Sus scrofa chromosome 18 genome assembly (NCBI Reference Sequence NC_010460). The position of all genes and their classification and functionality are reported. [file Table_2.pdf]

Supplementary Table S2. Description of the unrelated TRB genes in the *Sus scrofa* chromosome 18 genome assembly (NCBI Reference Sequence NC_010460). The position of all genes and their classification and functionality are reported.

**Gene classification**	**Functionality**	**Position** (complement)
MOXD2	F	7750229-7741199
TRY1	F	7722936-7715969
TRY2	F	7711199-7705814
TRY3	F	7696007-7691245
TRY5	F	7446624-7443380
EPBH6	F	7355665-7348703
