# Supplementary material for: Overview of the Germline and Expressed Repertoires of the TRB Genes in Sus scrofa
Source: Front Immunol. 2018 Nov 5;9:2526. doi: 10.3389/fimmu.2018.02526 (PMC6230588; doi:10.3389/fimmu.2018.02526)
Supplement: Supplementary Table S3 — Description of the Susscr TRBV pseudogenes. [file Table_3.PDF]

Supplementary Table S3. Description of the *Susscr* TRBV pseudogenes

**TRBV genes**	**Defective Leader**	**Frameshift**	**Stop codon**	**Defective splice sites**	**Defective RSS**
TRBV2-1		●			
TRBV2-2		●			
TRBV2-3				●	
TRBV2-5		●		●	
TRBV4-2		●			
TRBV5-2		●			
TRBV6			●		
TRBV8		●	●		
TRBV12-2		●			
TRBV22		●		●	
TRBV23	●	●			
TRBV28					●
