# Supplementary material for: Overview of the Germline and Expressed Repertoires of the TRB Genes in Sus scrofa
Source: Front Immunol. 2018 Nov 5;9:2526. doi: 10.3389/fimmu.2018.02526 (PMC6230588; doi:10.3389/fimmu.2018.02526)
Supplement: Supplementary Table S4 — Output file of the program RepeatMasker. Summary of the repeat content of pig TRB locus is shown. The GC levels are also indicated. [file Table_4.PDF]

Supplementary Table S4. Output file of the program RepeatMasker. Summary of the repeat content of pig TRB locus is shown. The GC levels are also indicated.

```
==================================================
file name: PIG
sequences:            1
total length:     402496 bp
GC level:         46.41 %
bases masked:     160358 bp (39.84 %)
==================================================
               number of      length   percentage
               elements*    occupied  of sequence
--------------------------------------------------
SINEs:               294       63005 bp   15.65 %
      Alu/B1           0           0 bp    0.00 %
      MIRs            34        4831 bp    1.20 %

LINEs:               130       58848 bp   14.62 %
      LINE1          105       54559 bp   13.56 %
      LINE2           21        3615 bp    0.90 %
      L3/CR1           4         674 bp    0.17 %
      RTE              0           0 bp    0.00 %

LTR elements:         74       28042 bp    6.97 %
      ERVL            15        4784 bp    1.19 %
      ERVL-MaLRs      21        6490 bp    1.61 %
      ERV_classI      36       16488 bp    4.10 %
      ERV_classII      0           0 bp    0.00 %

DNA elements:         27        5354 bp    1.33 %
      hAT-Charlie     10        1396 bp    0.35 %
      TcMar-Tigger     3         541 bp    0.13 %

Unclassified:          0           0 bp    0.00 %

Total interspersed repeats:   155249 bp   38.57 %


Small RNA:           261       58246 bp   14.47 %

Satellites:            0           0 bp    0.00 %
Simple repeats:      120        4178 bp    1.04 %
Low complexity:       20         830 bp    0.21 %
==================================================

* most repeats fragmented by insertions or deletions
  have been counted as one element
```
