# Supplementary material for: Overview of the Germline and Expressed Repertoires of the TRB Genes in Sus scrofa
Source: Front Immunol. 2018 Nov 5;9:2526. doi: 10.3389/fimmu.2018.02526 (PMC6230588; doi:10.3389/fimmu.2018.02526)
Supplement: Supplementary Table S5 — CDR3 nucleotide and predicted amino acid sequences retrieved from the TRB cDNA clones. CDR3-IMGT sequences are shown from codon 105 (the codon after the 2nd-CYS 104 of the V-REGION) to codon 117 (the codon before J-PHE 118 of the J-REGION) according to the unique numbering (25). The CDR3 nucleotide/amino acid sequence, and the classification of the TRBV, TRBD and TRBJ genes of each clone are also listed. Nucleotides of the 3'V-REGION and of the 5'J-REGION are indicated in uppercase letters. The sequences considered to present recognizable TRBD genes are indicated in bold lowercase letters and the nucleotide substitutions or insertions are underlined. Nucleotides that cannot be attributed to any V, D or J region (N-nucleotides), are indicated in lower cases on the left and on the right sides of the TRBD regions. The Accession number of the clones is reported. [file Table_5.PDF]

upplementary Table S5. CDR3 nucleotide and predicted amino acid sequences retrieved from the TRB cDNA clones DR3-IMGT sequences are shown from codon 105 (the codon after the 2nd-CYS 104 of the V-REGION) to codon 11 the codon before J-PHE 118 of the J-REGION) according to the unique numbering (Lefranc et al., 2003). The CDR ucleotide/amino acid sequence, and the classification of the TRBV, TRBD and TRBJ genes of each clone are als isted. Nucleotides of the 3'V-REGION and of the 5'J-REGION are indicated in uppercase letters. The sequence onsidered to present recognizable TRBD genes are indicated in bold lowercase letters and the nucleotide substitution r insertions are underlined. Nucleotides that cannot be attributed to any V, D or J region (N-nucleotides), are indicate n lower cases on the left and on the right sides of the TRBD regions. The Accession number of the clones is reported.

clone	TRBV gene	CDR3-IMGT	TRBD gene	TRBJ gene	CDR3 length (aa)
AY690915	21	GCCAGCAGCAAAGCgcggtggccgcctttgGAGACCCAGTAC<br>A S S K A R W P P L E T Q Y	-	3-5	14
AY690916	4-3/4	GCCAGCAGTAGgc**gggacag**acagTATAAC<br>A S S R R **D** **R** Q Y N	1	1-2	10
AY690917	20-3	GGTGCTAGgaaTTCCTATAATTCGCCCCTCCAC<br>G A R N S Y N S P L H	-	1-7	11
AY690918	1	ACCTGCAGTcgcaggAGTCAAAACACTCAGCAC<br>T C S R R S Q N T Q H	-	3-4	11
AY690919	27	GCCAGC**ggccaggggg**acGAGACCCAGTAC<br>A S **G** **Q** **G** D E T Q Y	1	3-5	10
AY690920	4-3/4	GCCAGCAGTAGA**cagggggg**aaaactccatAAC<br>A S S R **Q** **G** **G** K L H N	1	1-2	11
AY690921	4-3/4	GCCAGCAGTgtagggcccatAAACACTGAAGTCTTC<br>A S S V G P I N T E V F	-	1-1	12
AY690922	4-1	GCCAGCAGTa**gggacag**aagccttaAAAACATTCAGTAT<br>A S S R **D** **R** S L K N I Q Y	1	2-3	13
AY690923	4-1	GCCAGCAaggcagatgggGACTATAAC<br>A S K A D G D Y N	-	1-2	9
AY690924	7-1	GCCAGCAGCccc**gggaca**TCAGAGACCCAGTAC<br>A S S P **G** **T** S E T Q Y	1	3-5	11
AY690925	7-1	GCCAGCAGCTcccgttttgtccccgtaaagaattgTGACTATAAC<br>A S S S R F V P V K N C D Y N	-	1-2	15
AY690926	7-1	GCCAGCggtt**accggg**atactaaTGACTATAAC<br>A S G Y **R** D T N D Y N	1	1-2	11
AY690927	4S7	GCCAGCAGcccgg**caggg**aaagaCTTTGGAGACACCTACTTT<br>A S S P A **G** K D F G D T Y F	1	1-3	14
AY690928	7-1	GCCAGCAGCcca**ggacagg**ATTCGCCCCTCCAC<br>A S S P **G** **Q** D S P L H	1	1-7	11
AY690929	7-2	GCCAGCt**tggggg**ccacaataaagGACTATAAC<br>A S L **G** A T I K D Y N	3/2	1-2	11
AY690930	5-1	GCCAGCAGCCTatctcc**gggacagg**tagaGGGGCAGCTGTAT<br>A S S L S P **G** **Q** V E G Q L Y	1	2-2	14
AY690931	25	GCCAGCAGacccgcc**gggacagg**AGAGACCCAGTAC<br>A S R P A **G** **T** **G** E T Q Y	1	3-5	12
AY690932	20-1	GCTGCTAGTGAtcatc**gagctatggggggggg**gccccatTCACAGACCCAGTAC<br>A A S D H R **A** **M** **G** **G** G P H S Q T Q Y	3	2-4	18
AY690933	4-1	GCCAGCAacaggtacctggtccg**tgggg**ttcgggATTCGCCCCTCCAC<br>A S N R Y L V R **G** V R D S P L H	3/2	1-7	16
AY690934	20-3	GGTGCTAGTagatcgggctatcgcgATGAGCAGATT<br>G A S R S G Y R D E Q I	-	2-6	12
AY690935	25	GCCAGCAaggaa**gacagggggg**aAAACACGGGGCAGCTGTAT<br>A S K E **D** **R** **G** E N T G Q L Y	1	2-2	14
AY690936	6	GCCAGCaaacacaccacattt**cgggg**aGACTATAAC<br>A S K H T T F **R** **G** D Y N	1	1-2	12
AY690937	20-3	GGTGCTAGTGAt**tggggggg**cctCACTGAAGTCTTC<br>G A S D **W** **G** **G** L T E V F	3/2	1-1	12
AY690938	25	GCCAGCAGTGttctcctgtacagttacGGAGACACCTACTTT<br>A S S V L L Y S Y G D T Y F	-	1-3	14

AY690939	20-3	GGCGCTAGactgaaaccagacgggtttcCAAACACTGAAGTCTTC<br>G A R L K P D G F P N T E V F	-	1-1	15
AY690940	12-1	GCCAGCAGTGTTG**atggggggggg**ttggagacacacaACAATGACCTGCAT<br>A S S V D **G** **G** **G** L E T H N N D L H	3	2-1	17
AY690941	4-1	GCCAGCAGTc**ggacgggg**ttcggcacacAGACCCAGTAC<br>A S S R **T** **G** F G T Q T Q Y	1	3-5	13
AY690942	7-1	GCCAGCtcgtataACTCACAGACCCAGTAC<br>A S S Y N S Q T Q Y	-	2-4	10
AY690943	4S7	GCCAGCAGTAGAGAagac**ggggggg**ataACAATGACCTGCAT<br>A S S R E D **G** **G** D N N D L H	1/3/2	2-1	14
AY690944<br>AY690946	12-1	GCCAGCAGTGTTGGccgtcCGGGGCAGCTGTAT<br>A S S V G R P G Q L Y	-	2-2	11
AY690945	12-1	GCCAGCAGTGTTacc**gggacaggggtggc**acCAGACCCTCTGTAT<br>A S S V T **G** **T** **G** **V** A P D P L Y	1	3-3	15
AY690947	11	GCCAc**ccggggtggc**gcggagggagacacctacTTT<br>A T **R** **G** **G** A E G D T Y F	1	1-1	12
AY690948	5-1	GCCAGCAGCCcccgcgcgagacctAGCACAGACCCTCTGTAT<br>A S S P R A R P S T D P L Y	-	3-3	14
AY691000	4-5	GCCAGCAGcc**gggac**ccccATAATTCGCCCCTCCAC<br>A S S R **D** P H N S P L H	1	1-7	12
AY691001	7-1	GCCAGCAGCTTAcgag**caggggg**tcGAGACACCTACTTT<br>A S S L R A **G** **G** R D T Y F	1	1-3	13
AY691002	29	AGCGCag**gggac**gggagCACTCAGCAC<br>S A G **D** G S T Q H	1	3-4	9
AY691003	15	GCCAGCAGCAGAGAtcttgcccgtcttcatggACAGACCCAGTAC<br>A S S R D L A R L H G Q T Q Y	-	2-4	15
AY691004	7-2	GCCAGCAGCTgg**acaggggggc**cgACATTCAGTAT<br>A S S W **T** **G** **G** A D I Q Y	1	2-3	12
AY691005	21	GCCAGCAGCAAggaga**gggac**g**gggac**acCTATGACTATAAC<br>A S S K E R **D** G **D** T Y D Y N	1-1	1-2	14
AY691006	15	GCCAGCAGCAGc**atgggg**cttctaTATGAGCAGATT<br>A S S S **M** **G** L L Y E Q I	3/2	2-6	12
AY691007	5-1	GCCAGCAGtctgg**gggacaggggggg**gacggggAAC<br>A S S L G **D** **R** **G** **G** T G N	1	1-2	12
AY691008	5-1	GCCAGCAGCCTGGc**cagggggg**cttaggGAAAAGCTCATT<br>A S S L A **R** **G** L R E K L I	1	1-5	13
AY691009	7-1	GCCAGCAGCTTAGTcggctccaaggACACTCAGCAC<br>A S S L V G S K D T Q H	-	3-4	12
AY691010	7-2	GCCAGCAGCTacatt**ggggaccg**cgtCTATGAGCAGATT<br>A S S Y I G **D** **R** V Y E Q I	1	2-6	13
AY691011	15	GCCAGCAGCAGAGAtcggcagacaTATGACTATAAC<br>A S S R D R Q T Y D Y N	-	1-2	12
AY691012	7-1	GCCAGCAGCTTAa**atggggggg**cctttgtTGAGCAGCAT<br>A S S L N **G** **G** A F V E Q H	3/2	3-1	13
AY691013	7-1	GCCAGCAGCTTAGgttcaggtttgcgTGAAAAGCTCATT<br>A S S L G S G L R E K L I	-	1-5	13
AY691014	7-2	GCCAGCAGCTacatt**ggggaccg**cgtCTATGAGCAGATT<br>A S S Y I G **D** **R** V Y E Q I	1	2-6	13
AY691015	2-4	GCCAGtacacatggc**agaggg**GACCCTCTGTAT<br>A S T H G **R** **G** D P L Y	1	3-3	11
AY691016	7-1	GCCAGCAGCTTAcagatttcatATTCAGAGACCCAGTAC<br>A S S L Q I S Y S E T Q Y	-	3-5	13
AY691017	4S7	GCCAccaaggATAAC<br>A T K D N	-	1-2	5
AY691018	5-1	GCCAGCAGCCTaataccaAACACTCAGCAC<br>A S S L I P N T Q H	-	3-4	10
AY691019	7-2	GCCAGCgttctag**caggg**aACAATGACCTGCAT<br>A S V L A **G** N N D L H	1	2-1	12
AY691020	19	GCCAGCAGa**ggacagggg**agagcgGAGACCCAGTAC<br>A S R **G** **Q** **G** R A E T Q Y	1	3-5	12
AY691021	7S4	GCCAGCAGt**gaatta**gcccgaaaAAACACGGGGCAGCTGTAT<br>A S S **E** **L** A R K N T G Q L Y	3/2	2-2	14
AY691022	7-1	GCCAGCAGCCTAGactcttcGAGCAACCAGGCGCAGCAT<br>A S S L D S S S N Q A Q H	-	1-6	13
AY691023	7S4	GCCAGCAGacccc**ggacaggg**agacta**cgggggggg**tcacaCTATGAGCAGATT<br>A S R P R **T** **G** R L **R** **G** **G** S H Y E Q I	1-1	2-6	18

AY691024	4S5	GCCAcctccgt**gggacaggggg**cataTATGAGCAGATT<br>A T S V **G** **Q** **G** **G** I Y E Q I	1	2-6	13
AY691025	7S4	GCCAGCAGCTTAGGAGGCACCTACTTT<br>A S S L G G T Y F	-	1-3	9
AY691026	7-1	GCCAGCAGCccacca**cggggcagggg**tatgACTGAAGTCTTC<br>A S S P P **R** **G** **R** **G** M T E V F	1	1-1	14
AY691027	7-2	GCCAGCAGCCTAtggacagaacgacgAGGGGCCACCCTGACC<br>A S S L W T E R R G A T L T	-	2-5	14
AY691028	7S4	GCCAGCAGCTTAGTagta**agctat**GACTATAAC<br>A S S L V V **S** **Y** D Y N	3/2	1-2	11
AY691029	7S4	GCCAGCAGCctgct**gggggg**tTATAAC<br>A S S L L **G** **G** Y N	1	1-2	9
AY691030	12-1	GCCAGCAGTGTTGGccgacaaTTTGGAGACACCTACTTT<br>A S S V G R Q F G D T Y F	-	1-3	13
AY691031	12-1	GCCAGCAGTGat**gggacagggg**ATGACTATAAC<br>A S S D **G** **T** **G** D D Y N	1	1-2	11
AY691032	12-1	GCCAGCAGgcga**gacagg**cgaGACTGTAAC<br>A S R R **D** **R** R D C N	1	1-2	10
AY691033	12-1	ACCAGCAGTGgt**accggg**GACACCTACTTT<br>T S S G **T** **G** D T Y F	1	1-3	10
AY691034	12-1	GCCAGCAGTcg**gggaca**atctgACATTCAGTAT<br>A S S R **G** **Q** S D I Q Y	1	2-3	11
AY691035	3	GCCAGCAGTTc**gacagg**cctCACTGAAGTCTTC<br>A S S S **T** **G** L T E V F	1	1-1	11
AY691036	5-2	GCCAGCAGCGcggagatcg**ggagctacgggggggg**cgtttgGGGGCAGCTGTAT<br>A S S A E I G **S** **Y** **G** **G** **G** V W G Q L Y	3	2-2	18
AY691037	21	GCCAGCAGCAAAattaggctacCCAATGAAAAGCTCATT<br>A S S K I R L P N E K L I	-	1-5	13
AY691038	5-2	GCCAGCAGCGAcggactaaac**caggg**cGACCTGCAT<br>A S S D G L N **Q** **G** D L H	1	2-1	12
AY691039	5-1	GCCAGCAGCCgagatctagACATTCAGTAT<br>A S S R D L D I Q Y	-	2-3	10
AY691040	5-2	GCCAGCAGCccggagttc**cgggg**tATTCAGTAT<br>A S S P E F **R** **G** I Q Y	1	2-3	11
AY691041	5-2	GCCAGCAGCGAAacctcaTCACAGACCCAGTAC<br>A S S E T S S Q T Q Y	-	2-4	11
AY691042	5-1	GCCAGCAGCCcgcgtttgagacccgca**gatcggg**aAAACACGGGGCAGCTGTAT<br>A S S P R L R P A **D** **R** E N T G Q L Y	1	2-2	18
AY691043<br>AY691047	7S3	GCCAGCAGCTTActagccttagacGAAGTCTTC<br>A S S L L A L D E V F	-	1-1	11
AY691044	4S6	GCCAGCAatcgggacgggtcCTATGACTATAAC<br>A S N R D G S Y D Y N	-	1-2	11
AY691045	7S4	GCCAGCAGCTTttctggcACAGGGGCCACCCTGACC<br>A S S F S G T G A T L T	-	2-5	12
AY691046	7S3	GCCAGCAGg**atgaggg**aaGACACCTACTTT<br>A S R **M** **R** E D T Y F	3/2	1-3	10
AY691048	7S4	GCCAGCAGCaccttag**gacag**actaatAAC<br>A S S T L **G** **Q** T N N	1	1-2	10
AY691049	7S4	GCCAGCAGtggggataag**acgggg**CAGCTGTAT<br>A S S G D K **T** **G** Q L Y	1	3-2	11
AY691050	7-1	GCCAGCAGCggtgac**gggac**tagacTTGGAGACACCTACTTT<br>A S S G D **G** **T** R L G D T Y F	1	1-3	14
AY691051	4-3/4	GCCAGCAGTAGAattcca**ggacaggggggg**cgcaggTGAAAAGCTCATT<br>A S S R I P **G** **Q** **G** **G** A G E K L I	1	1-5	16
AY691052	12-1	GCCAGCAGT**gacaggg**tggaTGGAGACACCTACTTT<br>A S S **D** **R** V D G D T Y F	1	1-3	12
AY691053	12-1	GCCAGtgagcta**cggggggg**ggtgtgggatcCCTATGAGCAGATT<br>A S E L **R** **G** **G** V W D P Y E Q I	-	2-6	15
AY691054	12-1	GCCAGCAGTGTTatc**ggggg**TGAGCAGATT<br>A S S V I **G** **G** E Q I	1/3/2	2-6	10
AY691055	12-1	GCCAGCAGT**gggggggg**cccggACATTCAGTAT<br>A S S **G** **G** **G** P D I Q Y	3	2-3	11
AY691056	12-1	GCCAGCAGTGTTGG**gctacggggg**AACATTCAGTAT<br>A S S V G **L** **R** **G** N I Q Y	3/2	2-3	12
AY691057	5-1	GCCAGCAGCCTGGgt**cgggggg**tggggTATGACTATAAC<br>A S S L G **R** **G** V G Y D Y N	1	1-2	13

AY691058	21	GCCAGCAGCccccgggc**gggacag**atAAACACTGAAGTCTTC<br>A S S P R A **G** **Q** I N T E V F	1	1-1	14
AY691059	5-2	GCCAGCAGCccgcttacccgggcgttAAACACTGAAGTCTTC<br>A S S P L T R A L N T E V F	-	1-1	14
AY691060	21	GCCAGCAGCAtaggCTATGACTATAAC<br>A S S I G Y D Y N	-	1-2	9
AY691061	5-2	GCCAGCAGCGAAGc**ggacaggg**atcatGCTGAGCAGCAT<br>A S S E A **D** **R** D H A E Q H	1	3-1	13
AY691062	5-2	GCCAGCAGCccc**gggaca**tcaAGCAACCAGGCGCAGCAT<br>A S S P **G** **T** S S N Q A Q H	1	1-6	13
AY691063	5-1	GCCAGCAGCtactacGAAAAGCTCATT<br>A S S Y Y E K L I	-	1-5	9
AY691064	5-1	GCCAGCAGCCcgaggacccccagCTCCTACAATGACCTGCAT<br>A S S P R T P S S Y N D L H	-	2-1	14
AY691065	7S4	GCCAGCAGCTTAGTactaacaact**gggggc**tatactgATGACTATAAC<br>A S S L V L T T **G** **G** Y T D D Y N	1	1-2	16
AY691066	7-1	GCCAGCAGCTTAGTaggtaccccc*gacagaaaAAC<br>A S S L V G T P X Q K N	-	1-2	12
AY691067	7-1	GCCAGCAGCTTcc**gggacaggggtggc**TATGACTATAAC<br>A S S F R **D** **R** **G** **G** Y D Y N	1	1-2	13
AY691068	7S4	GCCAGCAacttc**gagacagag**acCTATGACTATAAC<br>A S N F **E** **T** **E** T Y D Y N	1	1-2	11
AY691069	7S4	GCCAGCAGC**cagggg**caggccctgactttacgaTCGCCCCTCCAC<br>A S S **Q** **G** Q A L T L R S P L H	1	1-7	15
AY691070	7-1	GCCAGCAGCTTAtattgggatgaGGCCACCCTGACC<br>A S S L Y W D E A T L T	-	2-5	12
AY691071	7-1	GCCAGCAGCTTAttagct**acggggggg**ccacCCTATGAGCAGATT<br>A S S L L A **T** **G** **G** P P Y E Q I	1	2-6	15
AY691072	7-1	GCCAGCAGCccgat**ggacag**attCTATGAGCAGATT<br>A S S P M **D** **R** F Y E Q I	1	2-6	12
AY691073	12-1	GCCAGCAGTccc**acaggg**ttcg**acacgggg**CAGCTGTAT<br>A S S P **T** **G** F D **T** **G** Q L Y	1-1	3-2	13
AY691074	12-1	GCCAGCAGTccattcggc**ggggggggg**atagATGAGCAGATT<br>A S S P F G **G** **G** **G** I D E Q I	3	2-6	14
AY691075	5-1	GCCAGCAGCCc**ggggggc**gcggagtagATAATTCGCCCCTCCAC<br>A S S P **G** **G** R G V D N S P L H	1	1-7	15
AY691076	5-1	GCCAGCAGCCcgcttaaa**gggggg**aaCAACCAGGCGCAGCAT<br>A S S P L K **G** **G** N N Q A Q H	1/3/2	1-6	14
AY691077	5-2	GCCAGCAGCGAActgttaagcgccccccCAGCAT<br>A S S E L L S A P Q H	-	3-1	11
AY691078	4-1	GCCAGCAGTAGTt**gatagggggg**cgatcaATGACTATAAC<br>A S S S **D** **R** **G** A I N D Y N	1	1-2	13
AY691079	4-3/4	GCCAGCAactcactcctgcccCTATGACTATAAC<br>A S N S L L P Y D Y N	-	1-2	11
AY691080	4-3/4	GCCAGCAGctcccgggatccgggTGGAGACACCTACTTT<br>A S S S R D P G G D T Y F	-	1-3	13
AY691081	4-1	GCCAGCAGTAcaccg**cggggggg**gaCCTATGAGCAGATT<br>A S S T P **R** **G** **G** T Y E Q I	1	2-6	13
AY691082	7-1	GCCAGCAGCTTggagaTAACAGGGGCCACCCTGACC<br>A S S L E I T G A T L T	-	2-5	12
AY691083	7-1	GCCAGCAGCcgtatca**acaggg**atc**atggg**AATGCGGCACAGCTGTAC<br>A S S R I N **R** D H **G** N A A Q L Y	1-3/2	3-2	16
AY691084	4-3/4	GCCAGCgccgat**gggac**caaattgaatcccctCAATGAAAAGCTCATT<br>A S A D **G** **T** K L N P L N E K L I	1	1-5	16
AY691085	4-1	GCCAGCAGTAGAGAtccgaccccgctattcaTGAATGCGGCACAGCTGTAC<br>A S S R D P T P L F M N A A Q L Y	-	3-2	17
AY691086	4-3/4	GCCAG**gggacaggggag**GACCCTCTGTAT<br>A R **G** **Q** **G** **E** D P L Y	1	3-3	10
AY691087	4-3/4	GCCAGCAGTAGAG**atggggggg**acgtcgATGAGCAGATT<br>A S S R D **G** **G** D V D E Q I	3/2	2-6	13
AY691088	7-1	GCCAGCAGCTTAGa**ggggcaggg**taAAAACACTCAGCAC<br>A S S L E **G** **Q** **G** K N T Q H	-	3-4	13
AY691089	7-1	GCCAGCAGCTTAGTacCACAGACCCAGTAC<br>A S S L V P Q T Q Y	-	2-4	10
AY691090	12-1	GCCAGtatgaat**gacaggg**aaGAGACCCAGTAC<br>A S M N **D** **R** E E T Q Y	1	3-5	11

AY691091<br>AY691092	5-1	GCCAGCAGCCTcgcc**gggacag**tcaatAATTCGCCCCTCCAC<br>A S S L A **G** **T** V N N S P L H	1	1-7	14
AY691093	21	GCCAGCAGCAAAGCtttgggtatcGACACCTACTTT<br>A S S K A L G I D T Y F	-	1-3	12
AY691094	7-1	GCCAGCAGtccg**gagctat**aTTCCTATGAGCAGATT<br>A S S P **E** **L** Y S Y E Q I	3/2	2-6	12
AY691095	7-1	GCCAGCAtccccgccgcagcgtggAACATTCAGTAT<br>A S I P A A A W N I Q Y	-	2-3	12
AY691096<br>AY691114	12-1	GCCAGCAGTGTT**gggggg**ACAGGGGCCACCCTGACC<br>A S S V **G** **G** T G A T L T	1/3/2	2-5	12
AY691097	12-1	GCCAGCAGTGTTGGacc**gggacaggg**ccCCTATGAGCAGATT<br>A S S V G P **G** **Q** **G** P Y E Q I	1	2-6	14
AY691098	12-1	GCCAGCAGTGccca**acaggggg**ctgaccatCAT<br>A S S A Q **Q** **G** A D H H	1	nd	11
AY691099	12-1	GCCAGCAGTaggcatacaGCTGAGCAGCA<br>A S S R H T A E Q H	-	3-1	10
AY691101	4-3/4	GCCAGCAGTAact**tggggg**ACGCTGAGCAGCAT<br>A S S N L **G** D A E Q H	3/2	3-1	11
AY691102	7-1	GCCAGCAGCCTA**acagga**tgggATTCGCCCCTCCAC<br>A S S L **T** **G** W D S P L H	1	1-7	12
AY691103	4-3/4	GCCAGCAGgaggct**acaggag**tCAGACCCTCTGTAT<br>A S R R L **Q** **E** S D P L Y	1	3-3	12
AY691104	4-3/4	GCCAGCAGcc**caggg**tggtccaCACAGACCCAGTAC<br>A S S P **G** W S T Q T Q Y	1	2-4	12
AY691105	4-3/4	GCCAGCAGTAGtgatga**gggacagggg**GACTATAAC<br>A S S S D E **G** **Q** **G** D Y N	1	1-2	12
AY691106	4-3/4	GCCAGCAGTAtga**gacagg**agAACACTCAGCAC<br>A S S M R **Q** E N T Q H	1	3-4	11
AY691107	4-3/4	GCCAGCAGTAGAGAttgggc**ggagct**tcgCACGGGGCAGCTGTAT<br>A S S R D W A **E** **L** R T G Q L Y	3/2	2-2	15
AY691108	12-1	GCCAGCAGcct**gggaca**GGCCACCCTGACC<br>A S S L **G** **Q** A T L T	1	2-5	10
AY691109	12-1	GCCAGCAGTGTT**gggggg**ACAGGGGCCACCCTGACC<br>A S S V **G** **G** T G A T L T	1/3/2	2-5	12
AY691110	12-1	GCCAGCAGTGTTccttgtttcg**tggcgggg**tgggAGACCCTCTGTAT<br>A S S V S L F R **G** **G** V G D P L Y	3/2	3-3	16
AY691111	12-1	GCCAGCAGTGTTa**gggac**ccccgGAGACCCAGTAC<br>A S S V R **D** P P E T Q Y	1	3-5	12
AY691112	12-1	GCCAGCAGTGgggccacc**ggacagggg**<br>A S S G A T **G** **Q** **G**	1	1-2	9
AY691113	12-1	GCCAGCAGT**atggggggg**cACATTCAGTAT<br>A S S **M** **G** **G** H I Q Y	3/2	2-3	10
AY691115	12-1	GCCAGCAGTGTT**GGgac**tgactactATTCAGAGACCCAGTAC<br>A S S V **G** **T** D Y Y S E T Q Y	1	3-5	14
AY691116	12-1	GCCAGC**ggacaggttgg**GACCAATGAAAAGCTCATT<br>A S **G** **Q** **V** **G** T N E K L I	1	1-5	12
AY691117	5-2	GCCAGCAGCccccgggcaagcTCCTATGAGCAGATT<br>A S S P R A S S Y E Q I	-	2-6	12
AY691118	5-1	GCCAGCAGCCTatataccccgGAGACCCAGTAC<br>A S S L Y T P E T Q Y	-	3-5	11
AY691119	5-1	GCCAGCAGCCaaccaaaatCAAACACTGAAGTCTTC<br>A S S Q P K S N T E V F	-	1-2	12
AY691120	5-2	GCCAGCAGCGAAGAcctcagtcgcGCGGCACAGCTGTAC<br>A S S E D L S R A A Q L Y	-	3-2	13
AY691121	5-1	GCCAGCAGCCTa**agctatggg**ctccCCTATGAGCAGATT<br>A S S L **S** **Y** **G** L P Y E Q I	3/2	2-6	13
AY691122	5-2	GCCAGCAccccgtcg**ggagc**ccaaattgcGCAT<br>A S T P S **G** **A** Q I A H	3/2	nd	11
AY691123	5-1	GCCAGCAGCCTGtcctcgctgagagacatcagt<br>A S S L S S L R D I S	-	nd	11
AY691124	5S4	GCCAGCAGCcactcc**gggacaggg**tcggaaTATAAC<br>A S S H S **G** **T** **G** S E Y N	1	1-2	12
AY691125	21	GCCAGCAGCAAgcccc**gggacag**cggttggGACTATAAC<br>A S S K P R **D** S G W D Y N	1	1-2	13
AY691126	7-1	GCCAGCAGCTctc**ggacaggggggc**AGAGACCCAGTAC<br>A S S S R **T** **G** **G** A E T Q Y	1	3-5	13

AY691127	7-1	GCCAGCAGCTTAtcc**gggacagggGG**AGACACCTACTTT<br>A S S L S **G T G G** D T Y F	1	1-3	13
AY691128	7-1	GCCAGCAtccatt**caggggg**CACTGAAGTCTTC<br>A S I H S **G G** T E V F	1	1-1	11
AY691129	7S4	GCCAGCAGCTTAGTggggg**gctacgggggggg**AAACATTCAGTAT<br>A S S L V G G **Y G G G** N I Q Y	3	2-3	15
AY691130	12-1	GCCAGCAGTGTTGa**gggggagg**TGAGCAGATT<br>A S S V E **G G G** E Q I	3	2-6	11
AY691131	12-1	GCCAGCAGTGagcta**cgggggg**acGAGCAGATT<br>A S S E L **R G** D E Q I	1	2-6	11
AY691132	12-1	GCCAGCAGTGTc**gggacaggg**TATGAGCAGATT<br>A S S V **G T G** Y E Q I	1	2-6	11
AY691133	12-1	GCCAGCAGTGggACTGAAGTCTTC<br>A S S G T E V F	-	1-1	8
AY691134	12-1	GCCAGCAGTGT**atggggggg**CTATGACTATAAC<br>A S S V **W G G** Y D Y N	3/2	1-2	11
AY691135	12-1	GCCAGCAGTGT**gggaca**aTCAGAGACCCAGTAC<br>A S S V **G Q** S E T Q Y	1	3-5	11
AY691136	12-1	GCCAGCAGTc**gggacag**CTATGACTATAAC<br>A S S R **D** S Y D Y N	1	1-2	10
AY691137	12-1	GCCAGCAGT**gggaca**GCAAACACTGAAGTCTTC<br>A S S **G T** A N T E V F	1	1-1	11
AY691138	12-1	GCCAGCAGTGTTGGatcctcc**gggaCA**GAGACCCAGTAC<br>A S S V G S S **G T** E T Q Y	1	3-5	13
AY691139	12-1	GCCAGtc**ggacgggg**AACACTCAGCAC<br>A S R **T G** N T Q H	1	3-4	9
AY691140	12-1	GCCAGCAGTcc**gagctat**ttcGCGGCACAGCTGTAC<br>A S S P **S Y** F A A Q L Y	3/2	3-2	12
AY691141	5-1	GCCAGCAGCCcaactccgtccAATTCGCCCCTCCAC<br>A S S P T P S N S P L H	-	1-7	12
AY691142	21	GCCAGCAGCAAAG**caggg**attaATAATTCGCCCCTCCAC<br>A S S K A **G** I N N S P L H	1	1-7	13
AY691143	5-1	GCCAGCAGCCTGGgacgcgtccCTGAGCAGCAT<br>A S S L G R V P E Q H	-	3-1	11
AY691144	5-1	GCCAGCAGCCctcg**acgggg***TTAACAGGGGCCACCCTGACC<br>A S S P R **R** X L T G A T L T	1	2-5	14
AY691145	5-1	GCCAGCAGCacc**gacagag**CTGAAGTCTTC<br>A S S T **D R** A E V F	1	1-2	10
AY691146	5-1	GCCAGCAGCgaagg**ggagct**cgtaTCCTATGAGCAGATT<br>A S S E G **E L** V S Y E Q I	3/2	2-6	13
AY691147	5-2	GCCAGCAGCGAAGtggccactcATTCAGAGACCCAGTAC<br>A S S E V A T H S E T Q Y	-	3-5	13
AY691148	5-1	GCCAGCAGCCgat**acaggggggc**gcagatggaatACAGACCCAGTAC<br>A S S R Y **R G G** A D G I Q T Q Y	1	2-4	16
AY691149	21	GCCAGCAGCccaa**atggggggg**tcctaAATGACCTGCAT<br>A S S P N **G G** V L N D L H	3/2	2-1	13
AY691150	4-1	GCCAGCAGTAcaccg**cgggggggg**aCCTATGAGCAGATT<br>A S S T P **R G G** T Y E Q I	1	2-6	13
AY691151	7-1	GCCAGCAGCTTggg**cagggg**ataCTATGAGCAGATT<br>A S S L G **R G** Y Y E Q I	1	2-6	12
AY691152	4-1	GCCAGCAGTActccgggg**aggaggg**aAGAGACCCAGTAC<br>A S S T P G **R R** E E T Q Y	1	3-5	13
AY691153	5-1	GCCAGCAGCCTGttagggcaAACAGGGGCCACCCTGACC<br>A S S L L G Q T G A T L T	-	2-5	13
AY691154	4-3/4	GCCAGCAGTAGAG**gggac**ttcAACACTGAAGTCTTC<br>A S S R G **D** F N T E V F	1	1-1	12
AY691155	4-1	GCCAGCAGc**cgcggaggg**AATTCGCCCCTCCAC<br>A S S **R G G** N S P L H	1	1-7	11
AY691156	4-3/4	GCCAGCAGattgga**acagggg**AATGAAAAGCTCATT<br>A S R L E **Q G** N E K L I	1	1-5	12
AY691157	7-2	GCCAGCAGCCTAa**gggac**AGCACAGACCCTCTGTAT<br>A S S L R **D** S T D P L Y	1	3-3	13
AY691158	5-1	GCCAGCAGCtggtggaCTCAGGAGCGGTAT<br>A S S W W T Q E R Y	-	3-7	10
AY691159	4-1	GCCAGCAGTccgtaccccgaagggcCCTATGAGCAGATT<br>A S S P Y P E G P Y E Q I	-	2-6	13

AY691160	15	GCCAGCAGCAGAGAcccggaatCAAACACTGAAGTCTTC<br>A S S R D P E S N T E V F	-	1-1	13
AY691161	7-2	GCCAGCAGCTTAaaaAGCACAGACCCTCTGTAT<br>A S S L K S T D P L Y	-	3-3	11
AY691162	7-2	GCCAGCAGCTTcggggtcggGACCAATGAAAAGCTCATT<br>A S S F G V G T N E K L I	-	1-5	13
AY691163	2-4	GCCAGCAGCTCtt**acagg**GAGCAGATT<br>A S S S Y **R** E Q I	1	2-6	9
AY691164	12-1	GCCAGCgcgaccttagggCAAAACACTCAGCAC<br>A S A T L G Q N T Q H	-	3-4	11
AY691165	7-1	GCCAGCAGCctcggccggacCACAGACCCTCTGTAT<br>A S S L G R T T D P L Y	-	3-3	12
AY691166	4-3/4	GCCAGCAGTAGcatt**agctat**GACTATAAC<br>A S S S I **S Y** D Y N	3/2	1-2	10
AY691167	15	GCCAGCAGC**atggggggggg**ccccGCACAGACCCTCTGTAT<br>A S S **M G G G** P R T D P L Y	3	3-3	14
